# Endothelial deletion of *Wt1* disrupts coronary angiogenesis and myocardium development

**DOI:** 10.1242/dev.201147

**Published:** 2023-03-27

**Authors:** Marina Ramiro-Pareta, Claudia Müller-Sánchez, Rosa Portella-Fortuny, Carolina Soler-Botija, Alejo Torres-Cano, Anna Esteve-Codina, Antoni Bayés-Genís, Manuel Reina, Francesc X. Soriano, Eloi Montanez, Ofelia M. Martínez-Estrada

**Affiliations:** ^1^ Celltec-UB, Department of Cell Biology, Physiology, and Immunology, Faculty of Biology, University of Barcelona, Barcelona 08028, Spain; ^2^ Institute of Biomedicine (IBUB), University of Barcelona, Barcelona 08028, Spain; ^3^ ICREC (Heart Failure and Cardiac Regeneration) Research Program, Health Science Research Institute Germans Trias i Pujol (IGTP), Can Ruti Campus, Badalona 08916, Spain; ^4^ CIBERCV, Instituto de Salud Carlos III, Madrid 28029, Spain; ^5^ CNAG-CRG, Centre for Genomic Regulation, Barcelona Institute of Science and Technology and Universitat Pompeu Fabra, Barcelona 08028, Spain; ^6^ Cardiology Service, Germans Trias i Pujol University Hospital, Badalona 08916, Spain; ^7^ Department of Medicine, UAB, Barcelona 08193, Spain; ^8^ Institut de Neurociències, Universitat de Barcelona, Barcelona 08028, Spain; ^9^ Department of Physiological Sciences, Faculty of Medicine and Health Sciences, University of Barcelona, L'Hospitalet de Llobregat 08907, Spain

**Keywords:** WT1, Heart development, Coronary endothelial cells, Blood vessel formation, Angiogenesis

## Abstract

*Wt1* encodes a zinc finger protein that is crucial for epicardium development. Although WT1 is also expressed in coronary endothelial cells (ECs), the abnormal heart development observed in *Wt1* knockout mice is mainly attributed to its functions in the epicardium. Here, we have generated an inducible endothelial-specific *Wt1* knockout mouse model (*Wt1KO*^ΔEC^). Deletion of *Wt1* in ECs during coronary plexus formation impaired coronary blood vessels and myocardium development. RNA-Seq analysis of coronary ECs from *Wt1KO*^ΔEC^ mice demonstrated that deletion of *Wt1* exerted a major impact on the molecular signature of coronary ECs and modified the expression of several genes that are dynamically modulated over the course of coronary EC development. Many of these differentially expressed genes are involved in cell proliferation, migration and differentiation of coronary ECs; consequently, the aforementioned processes were affected in *Wt1KO*^ΔEC^ mice. The requirement of WT1 in coronary ECs goes beyond the initial formation of the coronary plexus, as its later deletion results in defects in coronary artery formation. Through the characterization of these *Wt1KO*^ΔEC^ mouse models, we show that the deletion of *Wt1* in ECs disrupts physiological blood vessel formation.

## Teaser

**Highlighted Article:** A new mouse model in which *Wt1* expression is deleted in ECs reveals the crucial role of WT1 in coronary blood vessel development.

## INTRODUCTION

Endothelial cells (ECs) constitute the inner cellular layer of blood vessels. ECs perform a wide range of roles that are essential for organ formation and tissue homeostasis, including the transport of nutrients and metabolites to and from underlying tissues (Dejana et al., 2017). Besides these functions, ECs can also have unique and specialized roles to meet the distinct needs of the organs, and sites in which they are located (Dejana et al., 2017).

The adult heart is a highly vascularized organ with a specialized composition of cardiac ECs. In mice, heart vascularization starts around embryonic day (E) 11.5 when immature EC-progenitors derived from the sinus venosus (SV) and the endocardium begin to migrate and form an immature capillary plexus (Red-Horse et al., 2010; Wu et al., 2012). By E12.5, immature coronary ECs derived from the SV located in the subepicardial region proliferate and migrate into the compact myocardium, where they subsequently differentiate into intramyocardial coronary arteries and coronary veins (Lupu et al., 2020). Around E15.5, after blood flow is established, vascular remodelling occurs and the immature coronary endothelial plexus undergoes profound morphogenic changes that transform small vessels into mature, large-diameter arteries coated in vascular smooth muscle cells (VSMCs), which are visible by E17.5 (Lupu et al., 2020). The result of this plexus growth and remodeling is a mature coronary vasculature that is hierarchically arranged into arteries, capillaries and veins, a pattern that efficiently supports oxygenation of the myocardium (Lupu et al., 2020).

Coronary vasculature formation depends upon a series of tightly regulated steps in which ECs play an essential role (Luo et al., 2021). Defective coronary EC development leads to dramatic defects in coronary blood vessel formation and myocardium maturation (Chang et al., 2017; Gónzalez-Hernández et al., 2020; Luo et al., 2021; Rhee et al., 2018; Travisano et al., 2019; You et al., 2005). Coronary ECs also contribute to the rapid vascular growth of the neonatal heart and to the regeneration of new coronary blood vessels generated after myocardial infarction (MI) (Lu et al., 2021). Interestingly, this process depends on coronary ECs, as the new blood vessel structures formed in the injured myocardium arise mainly from pre-existing coronary ECs (He et al., 2017).

The WT1 transcription factor gene (*Wt1*) encodes a zinc-finger protein whose best-known function is its role as a transcription factor. WT1 also regulates gene expression post-transcriptionally through direct RNA binding (Bharathavikru et al., 2017; Hastie, 2017). WT1 plays a crucial role in the formation of several organs, including the heart (Hastie, 2017; Kreidberg et al., 1993; Moore et al., 1999). Recently, new studies have also highlighted its role in adult tissue homeostasis and repair(Ariza et al., 2019; Chau et al., 2011). For many years, it was thought that WT1 expression in the heart was confined to the epicardium and epicardium-derived cells (EPDCs). Thus, the embryonic lethality and cardiovascular defects observed in *Wt1* knockout mouse (*Wt1KO*) mice were mainly attributed to its functions in the epicardium (Martinez-Estrada et al., 2010; Moore et al., 1999). New evidence generated over the past decade has demonstrated the expression of WT1 in coronary ECs (Coppiello et al., 2015; Duim et al., 2015). Despite these findings, which suggest a role for WT1 in coronary ECs, our understanding of the cellular functions regulated by WT1 in coronary blood vessel development remains very limited (Cano et al., 2016).

In the present study, we generated and characterized an inducible EC-specific *Wt1KO* mouse model (*Wt1KO*^ΔEC^). Deletion of *Wt1* in coronary ECs during coronary plexus formation leads to dramatic defects in coronary blood vessel formation and myocardium development. RNA-sequencing (RNA-Seq) analysis of differentially expressed genes (DEG) in freshly isolated coronary ECs from control and mutant mice identified a set of DEGs that are modulated over the course of coronary EC development; more importantly, many of them regulate blood vessel formation. Many of the DEGs are involved in cell proliferation, migration and differentiation of coronary ECs, and, as a consequence, the aforementioned processes are affected in *Wt1KO*^ΔEC^ mice. The requirement of WT1 in ECs goes beyond the formation of the coronary plexus, as the later deletion of *Wt1* at more mature stages also impairs coronary blood vessel development. In summary, the data presented here demonstrate for the first time that endothelial deletion of *Wt1* disrupts physiological angiogenesis and myocardium development, and has a great impact in the molecular signature of coronary ECs.

## RESULTS

### Generation of a tamoxifen-inducible endothelial-specific *Wt1KO* mouse model

To validate previous findings reporting the expression of WT1 in coronary ECs, we performed WT1 immunostaining on heart sections at different stages of heart development (Coppiello et al., 2015; Duim et al., 2015). In line with previous results, immunostaining data demonstrated WT1 expression in coronary ECs of the coronary plexus from E12.5 onwards. Between E14.5 and E17.5, WT1 expression can be observed in an abundant number of subepicardial, intramyocardial and septal coronary ECs, but not in trabecular endocardium (Fig. S1).

To study whether WT1 is functionally important for coronary ECs and to overcome the embryonic lethality of conventional *Wt1KO* mice, we decided to generate an endothelial-specific *Wt1KO* mouse model. We used the *Pdgfb–iCreERT2* driver line, a widely used tamoxifen-inducible EC-specific Cre, which displays strong activity in coronary ECs during heart development and after MI (Claxton et al., 2008; Dube et al., 2017; Li et al., 2019; Luo et al., 2021; Travisano et al., 2019). First, *Pdgfb–iCreERT2* mice were crossed with the reporter *R26^mTmG/mTmG^* mice. In the *R26^mTmG/mTmG^* mouse model, before Cre-mediated recombination, membrane Tomato (mT) is expressed ubiquitously, and after Cre-mediated LoxP recombination, membranous GFP (mGFP) is expressed in Cre-positive cells (Muzumdar et al., 2007). Next, we administered tamoxifen to pregnant mice carrying *Pdgfb–iCreERT2; R26^mTmG/+^* mice during the early stages of coronary blood vessel formation (E11.5-E13.5) (Fig. S2A). Immunostaining analysis of GFP expression in the *Pdgfb–iCreERT2; R26^mTmG/+^* mice revealed robust mGFP expression in the developing heart at E15.5 days of development. mGFP^+^, CD31^+^ cells were located in the compact myocardium zone labelling both arteries and veins, but not in the ventricular endocardium or epicardium (Fig. S2B).

**Fig. 1.**
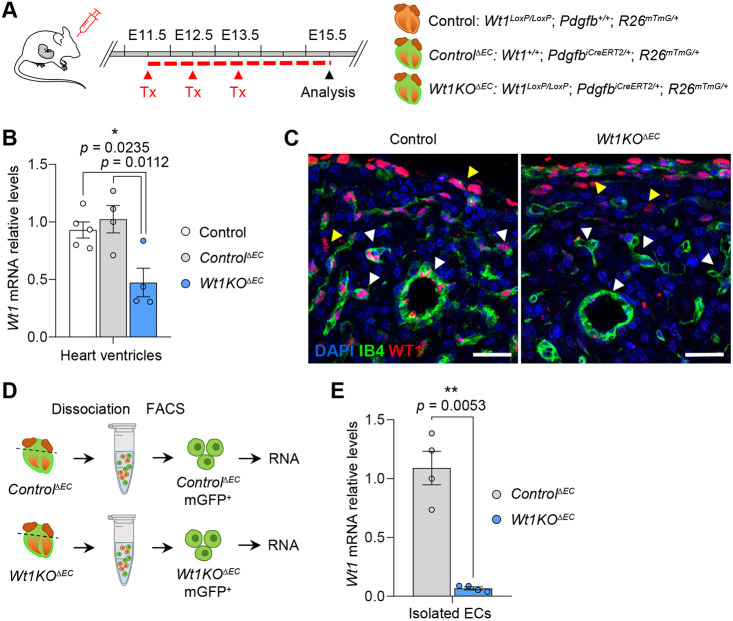
**Efficient deletion of *Wt1* in coronary ECs from *Wt1KO^ΔEC^* mouse model.** (A) Schematic illustration showing the tamoxifen administration scheme and the experimental strategy to obtain *Wt1KO^ΔEC^* and control mice. Pregnant mice carrying control and *Wt1KO^ΔEC^* embryos were administered tamoxifen in the early stages of coronary formation onset (E11.5-E13.5) and embryos were analysed at E15.5. (B) qRT-PCR analysis of *Wt1* expression in heart ventricles from *Wt1KO^ΔEC^* and control mice. Data are mean±s.e.m. (*n*=4 or 5). *P<0.05, one-way ANOVA followed by Tukey's post-hoc test. (C) Immunofluorescence staining for WT1 (red) and staining of IB4 (green) and nuclear DAPI (blue), using heart sections from control and *Wt1KO^ΔEC^* E15.5 mice. There is specific downregulation of WT1 expression in coronary ECs (white arrowheads) while its expression in the epicardium and EPDCs is not affected (yellow arrowheads). (D) EC isolation procedure from enzymatically digested *Control^ΔEC^* and *Wt1KO^ΔEC^* heart ventricles at E15.5. (E) qRT-PCR analysis of *Wt1* expression in mGFP^+^ FACS-isolated ECs from *Wt1KO^ΔEC^* and *Control^ΔEC^* hearts. A dramatic downregulation of *Wt1* expression is observed in ECs from *Wt1KO^ΔEC^* mice. Data are mean±s.e.m. (*n*=4). **P<0.01 (unpaired *t*-test with Welch's correction). Scale bars: 25 μm.

After verification of *Pdgfb–iCreERT2* activation using this tamoxifen scheme, we used this Cre line to delete the *Wt1* gene from coronary ECs by crossing *Wt1^LoxP/LoxP^* mice with *Pdgfb–iCreERT2* mice to generate *Wt1^LoxP/LoxP^; Pdgfb–iCreERT2* mice (*Wt1^LoxP/LoxP^; Pdgfb^iCreERT2/+^*). Given that the Pdgfb*–iCreERT2;R26^mTmG/+^* mouse model allowed visualization of coronary ECs in which Cre has efficiently recombined (mGFP^+^), a second mouse model was also generated: *Wt1^LoxP/LoxP^; Pdgfb^iCreERT2/+^; R26^mTmG/+^*. Daily administration of tamoxifen to pregnant mice during the coronary angiogenic phase (E11.5-E13.5) (Fig. 1A) caused a 50% loss of *Wt1* mRNA levels in heart ventricles from *Wt1^LoxP/LoxP^; Pdgfb^iCreERT2^; R26^mTmG/+^* mice at E15.5 (hereafter referred to as *Wt1KO^ΔEC^*) compared with *Wt1^LoxP/LoxP^; R26^mTmG/+^* and *Wt1^+/+^; Pdgfb^iCreERT2^; R26^mTmG/+^* mice (hereafter referred to as control and *Control^ΔEC^*, respectively) (Fig. 1B). Next, we performed immunostaining analysis of WT1 in heart sections from control and *Wt1KO^ΔEC^* mice at E15.5. The immunostaining data demonstrated the specific downregulation of WT1 in coronary endothelial cells in *Wt1KO^ΔEC^* hearts, whereas its expression in the epicardium was not affected (Fig. 1C). We then performed quantitative real-time PCR (qRT-PCR) analyses of *Wt1* expression in mGFP^+^ sorted ECs from *Wt1KO^ΔEC^* mice at E15.5, and observed a dramatic downregulation of *Wt1* levels in *Wt1KO^ΔEC^* compared with the *Control^ΔEC^* mice (Fig. 1D,E). Altogether, these findings demonstrate that *Wt1KO^ΔEC^* mice constitute a new mouse model in which *Wt1* is efficiently deleted in coronary ECs.

### Deletion of *Wt1* in coronary ECs during coronary plexus formation disrupts myocardium and coronary vessel development

**Fig. 2.**
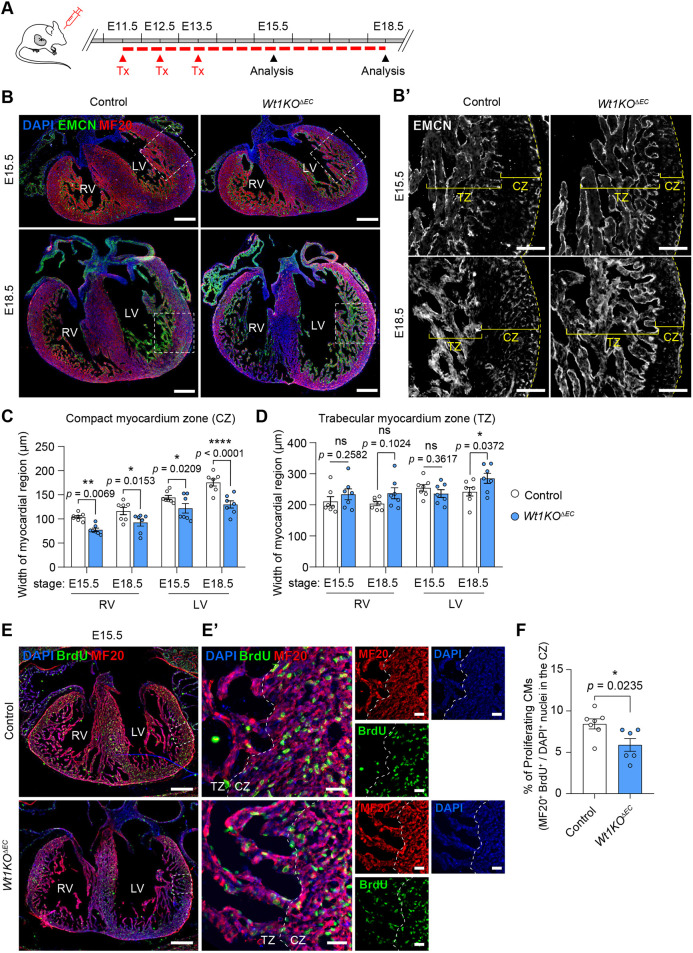
***Wt1KO^ΔEC^* mice display defects in myocardium development.** (A) Schematic illustration showing the experimental protocol strategy to obtain and analyse *Wt1KO^ΔEC^* mice. Pregnant mice were administered tamoxifen from E11.5 to E13.5 and embryos were analysed at E15.5 and E18.5. (B) Immunostaining for endomucin (EMCN, green) and sarcomeric myosin (MF20, red) on E15.5 and E18.5 heart sections from control and *Wt1KO^ΔEC^* mice. (B′) Area outlined in B, showing EMCN staining as an indicator of trabecular myocardium zone (TZ) versus the compact myocardium zone (CZ). (C,D) Quantification of CZ and TZ width in control and *Wt1KO^ΔEC^* mice confirms the significant effect of endothelial *Wt1* deletion on the CZ both at mid (E15.5) and late (E18.5) developmental stages. Data are mean±s.e.m. (*n*=7). *P<0.05, **P<0.01, ****P<0.0001 (two-way ANOVA). (E) E15.5 heart sections from control and *Wt1KO*^Δ*EC*^ mice treated with BrdU were immunostained using antibodies against sarcomeric myosin (MF20, red) and BrdU (green), and counterstained with DAPI (blue). (E′) Area outlined in E. (F) Quantitation of proliferative cardiomyocytes (CMs) (% BrdU^+^ MF20^+^/DAPI^+^) from control and *Wt1KO^ΔEC^* heart sections at E15.5. Data are mean±s.e.m (*n*=6-7). *P<0.05 (unpaired *t*-test). Scale bars: 250 µm in B,E; 50 µm in B′ and 25 µm in E′.

Until very recently, the abnormal cardiac vascularization and thinning of the ventricular myocardium observed in conventional *Wt1KO* mice have mainly been attributed to WT1 functions in epicardial cells, but little is known about the effect of *Wt1* deletion on coronary ECs in heart development (Martinez-Estrada et al., 2010; Moore et al., 1999; Velecela et al., 2019). We used the previously described tamoxifen strategy, harvesting embryos at E15.5 and E18.5, and performed double immunostaining against myosin heavy chain (MF20), to define the myocardium region, and endomucin, to delimit the compact from the trabecular myocardium, in control and *Wt1KO^ΔEC^* hearts (Fig. 2A,B). Morphological analyses of the hearts of *Wt1KO^ΔEC^* embryos showed that deletion of *Wt1* in coronary ECs severely impaired heart development. In *Wt1KO^ΔEC^* embryos, the compact myocardium was significantly thinner than in control embryos (Fig. 2B,C). Interestingly, the defects in compact myocardium development correlate with an expansion of the left ventricle trabecular myocardium (Fig. 2B,D). To ascertain whether this phenotype could be due to defects in cardiomyocyte proliferation, we assessed cell proliferation in control and *Wt1KO^ΔEC^* embryos. We injected 5-bromo-2′-deoxyuridine (BrdU) into tamoxifen-administered pregnant females at E15.5. Control and *Wt1KO^ΔEC^* embryos were then collected 2 h after the BrdU pulse, fixed and sectioned for later co-immunostaining for the proliferation marker BrdU together with MF20. Quantification of the immunostaining analysis revealed a reduction in the percentage of proliferating cardiomyocytes (%BrdU^+^/MF20^+^ cells) within the compact myocardium of *Wt1KO^ΔEC^* hearts compared with control littermates (Fig. 2E,F).

**Fig. 3.**
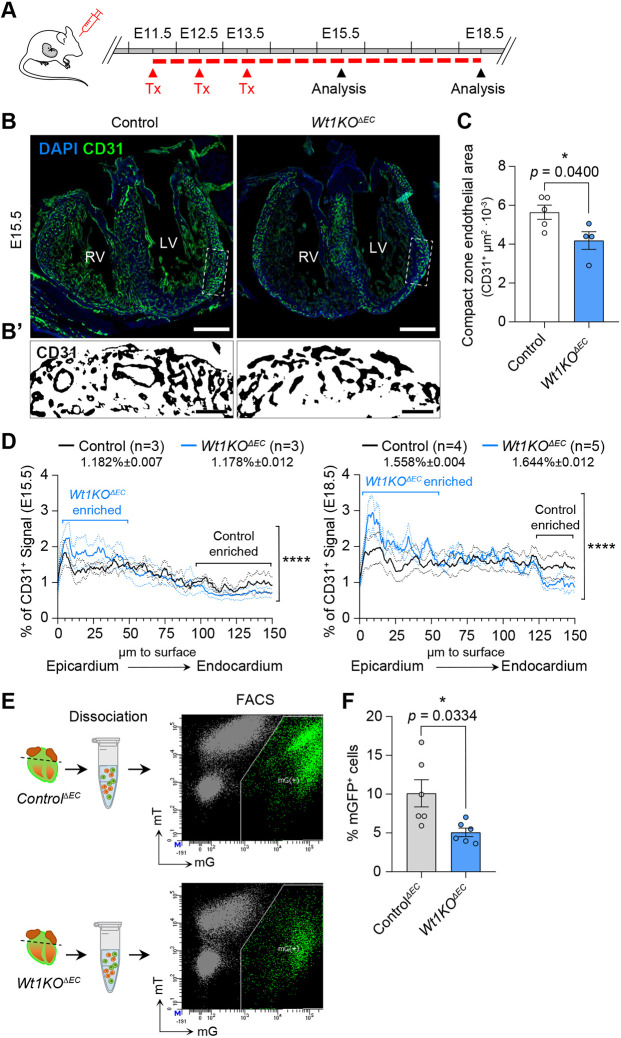
***Wt1KO^ΔEC^* mice display defects in coronary blood vessel development.** (A) Schematic illustration showing the experimental protocol strategy to obtain and analyse *Wt1KO^ΔEC^* mice. Pregnant mice were administered tamoxifen from E11.5 to E13.5, and embryos were analysed at E15.5 and E18.5. (B) Immunofluorescence staining for CD31 (green) and nuclear DAPI (blue), using heart sections from control and *Wt1KO^ΔEC^* at E15.5. (B′) Magnified images of CD31 staining from the areas outlined in B. (C) Quantitation of the CD31^+^ signal in the compact myocardium zone (CZ) of control and *Wt1KO^ΔEC^* hearts. Data are mean±s.e.m. (*n*=3). *P<0.05 (unpaired *t*-test). (D) Quantitation of CD31^+^ immunostaining localization from heart sections of control and *Wt1KO^ΔEC^* mice at E15.5 and E18.5, reported as a percentage of intensity within a particular bin against the distance from the epicardial surface of the heart. In the *Wt1KO^ΔEC^* heart, CD31^+^ staining is increased near the epicardium and reduced in deeper myocardium layers. Data are mean±s.e.m. (*n*=3, E15.5; *n*=4 or 5, E18.5). ****P<0.0001 (Mann–Whitney test). (E) EC isolation and representative FACS analysis of enzymatically digested *Control^ΔEC^* and *Wt1KO^ΔEC^* heart ventricles at E15.5. (F) Quantification of mGFP^+^ ECs from enzymatically digested *Control^ΔEC^* and *Wt1KO^ΔEC^* heart ventricles at E15.5. Data are mean±s.e.m. (*n*=6). *P<0.05 (unpaired *t*-test with Welch's correction). Scale bars: 250 µm in B; 50 µm in B′.

To rule out the possibility that these defects in the myocardium observed in mutant mice arose from defective epicardium formation, we analysed the expression of RALDH2, a marker of embryonic epicardium signature and a direct target of WT1 (Guadix et al., 2011). As expected, no difference in RALDH2 expression was observed in *Wt1KO^ΔEC^* mice compared with their control littermates, which correlates with the absence of recombination of the *Pdgfb–iCreERT2* line in the epicardium (Fig. S3). Abnormal coronary vessel development has previously been shown to lead to defects in myocardium development (D'Amato et al., 2016; Rhee et al., 2018; Travisano et al., 2019). Next, we decided to characterize coronary development in *Wt1KO^ΔEC^* embryos. We immunostained heart sections from control and *Wt1KO^ΔEC^* hearts obtained at E15.5 and E18.5 with CD31 antibody, a marker of ECs. Qualitative observations revealed that CD31^+^ vessels were present in both control and *Wt1KO^ΔEC^* hearts (Fig. 3A,B). However, large-calibre CD31^+^ vessels were scarce in the *Wt1KO^ΔEC^* hearts (Fig. 3B,B′). In addition, quantification of CD31^+^ staining in the compact myocardium revealed a decreased CD31^+^ area in *Wt1KO^ΔEC^* mice (Fig. 3C). Next, we decided to quantify localization of the CD31 signal in control and *Wt1KO^ΔEC^* mice. Quantification of CD31 localization also revealed an inappropriate accumulation of ECs near the epicardial surface in *Wt1KO^ΔEC^* hearts at E15.5 and E18.5 (Fig. 3D). The reduction in the number of ECs was validated using FACS analysis of enzymatically dissociated heart ventricles, which demonstrated a reduction in mGFP^+^ ECs in *Wt1KO^ΔEC^* mice (Fig. 3E,F).

We next investigated when these coronary blood vessel defects are first observed, and then analysed coronary plexus formation at E13.5. Close inspection of the coronary plexus using whole-mount GFP staining revealed that while the dorsal coverage of the endothelium was not affected, the ventral coverage was significantly reduced in *Wt1KO^ΔEC^* hearts by E13.5 (Fig. S4A-D). Interestingly, CD31 immunostaining of *Wt1KO^ΔEC^* sections at this stage demonstrated that the vascularzation of the compact myocardium was not altered when compared with control littermates (Fig. S4E,F). Likewise, at this stage the myocardium development was not affected in *Wt1KO^ΔEC^* hearts, indicating that the crosstalk between the ECs and cardiomyocytes is not compromised in *Wt1KO^ΔEC^* hearts at this stage (Fig. S4G-I). After observing the heart defects in *Wt1KO^ΔEC^* mice, we analysed their viability. Genotyping of embryonic mice showed a normal Mendelian ratio of *Wt1KO^ΔEC^* mice at E13.5, E15.5 and E18.5 (Table S1). Altogether, these data demonstrate that WT1 functions in ECs are required for proper coronary blood vessel development, and its deletion compromises myocardial development.

### *Wt1* regulates the transcriptomic signature of coronary ECs

**Fig. 4.**
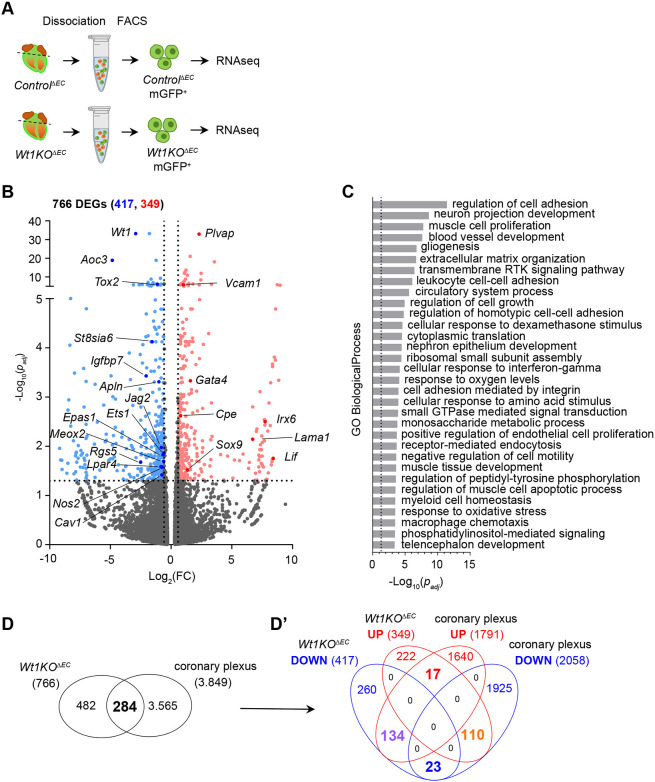
**WT1 regulates the transcriptomic profile of coronary ECs.** (A) Schematic illustration showing the experimental strategy to obtain coronary ECs for RNA-Seq from *Control^ΔEC^* and *Wt1KO*^Δ*EC*^ mice. E15.5 heart ventricles were enzymatically digested and mGFP^+^ ECs were FACS-isolated and processed for RNA extraction and sequencing. (B) Volcano plot of the 766 DEGs (Log2FC >|0.58|) identified by transcriptomic analysis of mGFP^+^ sorted cells from *Control^ΔEC^* versus *Wt1KO^ΔEC^* hearts. Significantly upregulated (349, red) and downregulated (417, blue) genes are represented in terms of significance, -Log10 (*P*-value). (C) GO enrichment analysis using DEGs from E15.5 *Wt1KO^ΔEC^*. Significantly up- and downregulated genes (*P*<0.05, and Log2FC>|0.58|) were used for this analysis; GO enrichment analysis was performed using a two-tailed hypergeometric test; the *P*-value was corrected using a Bonferroni step-down. Only Grouped GO Terms with *P*<0.05 are displayed (dotted line). (D) Venn diagram of a comparative analysis of deregulated genes from the transcriptomic profile of *Wt1KO*^Δ*EC*^ with DEGs during coronary EC remodeling. (D′) Up- and downregulated genes corresponding to both analyses. Numbers in bold indicate the total number of signatures common to the two analyses.

WT1 is a transcription factor that displays pleotropic functions through the direct regulation of several genes (Hastie, 2017). Next, we decided to gain an insight into the molecular events underlying *Wt1* deletion in coronary ECs. Taking advantage of our mouse model, we performed RNA-Seq of FACS isolated coronary ECs in which the Cre had efficiently recombined (mGFP^+^) from E15.5 *Control^ΔEC^* and *Wt1KO^ΔEC^* hearts (Fig. 4A). We found substantial changes in the transcriptomic profile, with 766 DEGs in the *Wt1KO^ΔEC^* ECs transcriptome (349 upregulated and 417 downregulated) (Table S2). Volcano plots summarizing this differential expression analysis are shown in Fig. 4B. Sequencing served as a quality control for our sorted strategy, as we observed a significant enrichment of EC genes such as *Pecam1*, *Fabp4*, *Cdh5*, *Cldn5*, *Col4a1*, *Col18a1* and *Flt1*, confirming the endothelial identity of the sorted cells. Moreover, gene signatures assigned to other cardiac populations, such as cardiomyocytes (*Nkx2*-5, *Actn2*, *Myh7*, *Mb* and *Tnnt2*), epicardial cells (*Upk1b*, *Upk3b*, *Clu* and *Dmkn*) and fibroblasts (*Col1a1*, *Gsn*, *Wif1*, *Dkk3*, *Mt1*, *Cthrc1* and *Acta2*), appeared at relatively low counts, thus validating our strategy of specific sorting of coronary ECs (Fig. S5A). Analysis of the sequencing data also demonstrated that, despite the strong phenotype observed in mutant mice, several canonical EC genes, such as *Pecam1*, *Cdh5* and *Cldn5*, were not modified by *Wt1* deletion (Fig. S5B-E). However, we found that several important genes in ECs, such as *Foxo1*, *Cav1*, *Sema5a*, *Sema6d*, *Apln*, *Cd109*, *Meox2*, *Jag2*, *Gli3* and *Igfbp7*, were downregulated in mutant cells (Table S2 and Fig. S5E). Next, we used gene ontology (GO) enrichment analysis to gain an insight into biological processes in the 766 DEGs. The analysis revealed the over-representation of some GO terms, such as ‘regulation of cell adhesion’, ‘blood vessel development’, ‘extracellular matrix organization’, ‘cytoplasmic translation’, ‘regulation of endothelial cell proliferation’ and ‘regulation of cell motility’ (Fig. 4C, full list in Table S3).

Next, we compared the transcriptomic profile of coronary ECs from the *Wt1KO^ΔEC^* mice generated in this study with the transcriptomic profile of developing coronary ECs published recently (Gónzalez-Hernández et al., 2020). This profile spans the active coronary angiogenic phase (E13.5) to the remodeling and maturation phase of embryonic coronary ECs (E17.5) (Gónzalez-Hernández et al., 2020). Interestingly, 284 DEGs in *Wt1KO^ΔEC^* belonged to genes expressed in coronary ECs during these different phases, of which 55.28% were downregulated and 44.72% were upregulated (Fig. 4D, Table S4). Out of these 157 downregulated genes in *Wt1KO^ΔEC^*, 85.35% were genes whose expression increased dynamically from E13.5 to E17.5. Conversely, 86.61% of the 127 upregulated genes in the *Wt1KO^ΔEC^* were genes abundantly expressed in coronary ECs in the angiogenic phase that decreased over the course of coronary EC maturation (Table S4).

Among the genes that were downregulated in *Wt1KO^ΔEC^* and increased dynamically over the course of coronary development were several that are important regulators of EC functions, such as *Apln*, *Cd109*, *Lpar4*, *Meox2*, *Jag2*, *Igfbp7* and *Aoc3*. Meanwhile, among the upregulated genes that should decrease their expression over the course of coronary development, we found, *Lif*, *Irx6*, *Egfl6*, *Sox9*, *Gata4* and *Lama1* (Table S4 and Fig. S5D). The comparison of both transcriptomic profiles suggests that mutant ECs fail to upregulate genes that belong to the more mature stages and remain at a more primitive stage (Fig. 4D, Fig. S5 and Table S4). Collectively, these analyses reveal that WT1 is a major regulator of the transcriptomic signature of coronary ECs cells and identify potential functional defects involved in the phenotype of *Wt1KO^ΔEC^* mice.

### Coronary EC proliferation and migration are affected in *Wt1KO^ΔEC^* mice

**Fig. 5.**
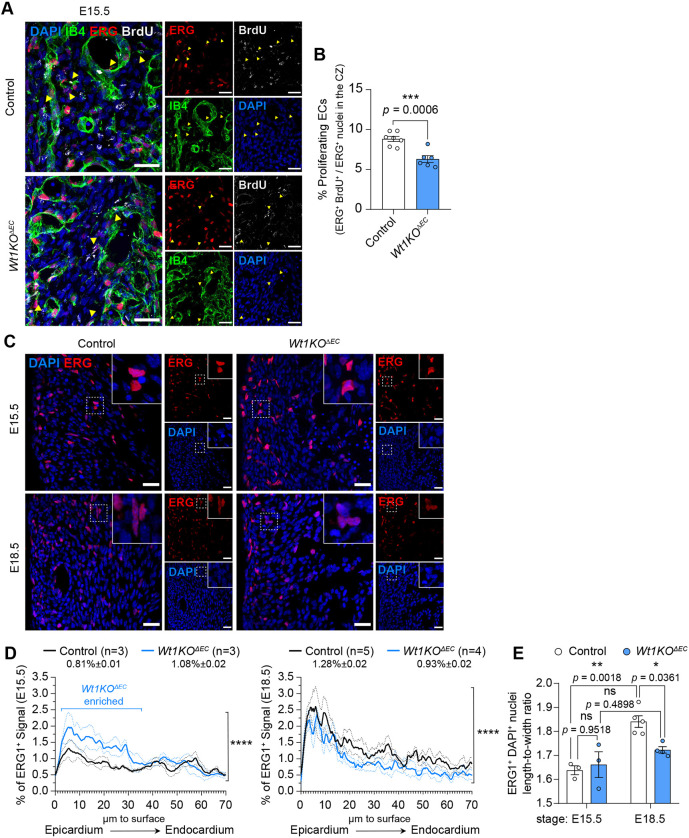
***Wt1KO* ECs exhibited impaired cell proliferation and migration.** (A) E15.5 heart sections from control and *Wt1KO*^Δ*EC*^ mice treated with BrdU were immunostained with antibodies against ERG (red), BrdU (white) and IB4 (green), and counterstained with DAPI (blue). (B) Quantitation of proliferative ECs (%BrdU^+^ERG^+^/ERG^+^) from control and *Wt1KO^ΔEC^*. Data are mean±s.e.m. (*n*=6 or 7). ***P<0.001, unpaired *t*-test. (C) Immunostaining for ERG (red) and nuclear DAPI staining (blue), using heart sections from control and *Wt1KO^ΔEC^* at the indicated stages. (D) Quantitation of ERG^+^ immunostaining localization, reported as a percentage of intensity within a particular bin against the distance from the epicardium surface. Data are mean±s.e.m. from control (*n*=3, E15.5; *n*=5, E18.5) and *Wt1KO^ΔEC^* hearts (*n*=3, E15.5; *n*=4, E18.5). ****P<0.0001 (Mann–Whitney test). (E) Quantitation of the length-to-width ratio of ERG^+^ nuclei. Data are mean±s.e.m. from control (*n*=3, E15.5; *n*=5, E18.5) and *Wt1KO^ΔEC^* hearts (*n*=3, E15.5; *n*=4, E18.5) of comparable compact myocardium fields *P<0.05, **P<0.01 (two-way ANOVA). Scale bars: 25 µm in A; 50 µm in C.

As many DEGs in the *Wt1KO^ΔEC^* transcriptome are involved in regulation of EC proliferation (Table S3 and Fig. S6A), and we found a reduction in coronary ECs in *Wt1KO^ΔEC^* mice, we investigated whether EC proliferation is affected in mutant mice. Control and *Wt1KO^ΔEC^* embryos previously administered with BrdU were immunostained using antibodies against ERG transcription factor – a marker of EC and anti-BrdU (Fig. 5A). Quantification of the immunostaining analysis revealed a reduction in the percentage of proliferating ECs (%BrdU^+^/ERG^+^ cells) within the compact myocardium of *Wt1KO^ΔEC^* hearts, compared with control littermates (Fig. 5B).

GO analysis of RNA-Seq data also revealed DEGs in *Wt1KO^ΔEC^* related to biological processes such as blood vessel development and cell motility (Fig. 4C and Fig. S6B). Thus, we decided to analyse whether cell migration and differentiation was affected in *Wt1KO^ΔEC^* hearts. To begin investigating the impact of *Wt1* deletion on the aforementioned processes, we immunostained sections from control and *Wt1KO^ΔEC^* hearts immediately after the onset of arterial flow (E15.5) and at a later stage (E18.5) against nuclear ERG to check for endothelial location (Fig. 5C). Localization of the ERG^+^ staining signal was quantified to confirm alteration of the EC migration (Fig. 5D). Quantification of ERG^+^ nucleus localization revealed an inappropriate accumulation of ECs near the epicardial surface in *Wt1KO^ΔEC^* hearts at E15.5 (Fig. 5D). ERG staining was also used to determine the nuclear shape/morphology of coronary ECs, as it has recently been demonstrated that there is a switch from a round to a spindle morphology over the course of EC maturation (Franco et al., 2016; Quijada et al., 2021). Next, the length-to-width ratio of ERG^+^ nuclei was quantified as an indicator of EC maturity: values close to 1 indicate a nuclear shape similar to a perfect circle, whereas higher numbers indicate nuclei elongation. A considerable change in nuclear shape was observed between E15.5 and E18.5 in control hearts, but the nuclei of *Wt1KO^ΔEC^* cells retained the shape of the more immature stage (Fig. 5E).

Having observed proliferation and migration defects in ECs from *Wt1KO^ΔEC^ in vivo*, we decided to examine the effect of *Wt1* deletion *in vitro*, without the potential side effects of altered heart development. We then generated primary cultures of ECs from tamoxifen-inducible *Wt1KO* mice. Analysis of CDH5 (VE-cadherin) and WT1 immunostaining revealed their endothelial identity and the expression of WT1 in these primary cultures (Fig. S7A). Moreover, qRT-PCR analysis confirmed that *Wt1* expression was significantly downregulated after 72 h of tamoxifen treatment (Fig. S7B). Next, performing transwell migration and BrdU proliferation assays, we found that, as in the *in vivo* situation, *in vitro* deletion of *Wt1* impairs these two processes in ECs (Fig. S7C,D). This demonstrates the cell-autonomous role of WT1 in the regulation of ECs proliferation and migration. Taken together, these data indicate that deletion of *Wt1* in coronary ECs impairs their proliferation, migration and maturation properties.

### Impairment of coronary EC differentiation in *Wt1KO^ΔEC^* mice

**Fig. 6.**
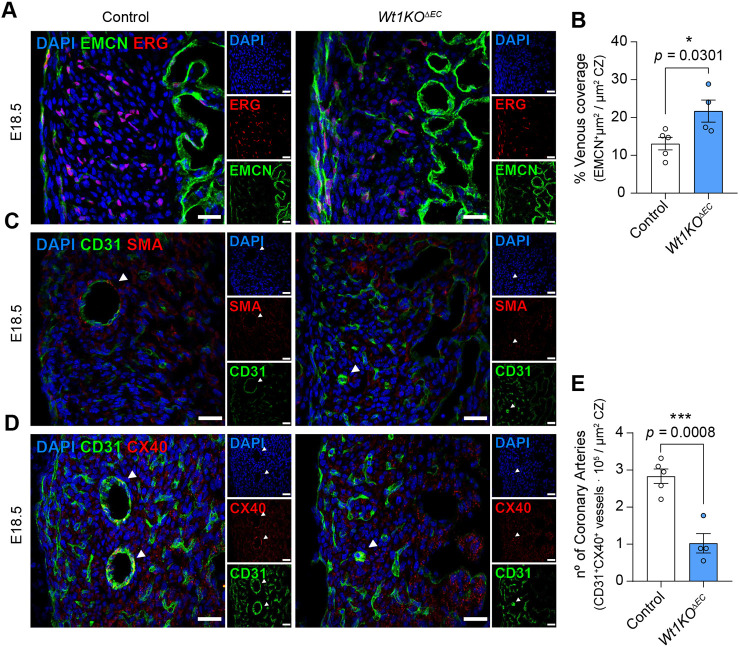
***Wt1KO^ΔEC^* hearts display an altered pattern of venous and arterial specification.** (A) Immunostaining for ERG (red), EMCN (green) and nuclear DAPI staining (blue), using heart sections from control and *Wt1KO^ΔEC^* E18.5 mice. Accumulation of EMCN^+^ vessels is observed in the subepicardial region of *Wt1KO^ΔEC^* hearts. (B) Quantitation of EMCN^+^ signal in the compact myocardium zone (CZ) shows increased percentage of venous coverage. Data are mean±s.e.m. *n*=5. *P<0.05 (unpaired *t*-test). (C) Immunofluorescence staining for PECAM (green) and SMA (red), and nuclear DAPI staining (blue), using heart sections from control and *Wt1KO^ΔEC^* mice at E18.5. Very few SMA-covered CD31^+^ vessels are observed in the CZ of *Wt1KO^ΔEC^* hearts. (D) Immunofluorescence staining of section from E18.5 hearts with antibodies against CD31 (green) and CX40 (red, arterial ECs) reveals small-calibre arteries in the *Wt1KO^ΔEC^*. (E) Quantitation of CX40^+^CD31^+^ vessels within the CZ. Data are mean±s.e.m. *n*=4 or 5. ***P<0.001 (unpaired *t*-test). Scale bars: 25 µm.

Coronary vessel differentiation requires re-specification of immature ECs towards arterial cell fate in deeper myocardium and towards venous cell fate in subepicardial myocardium (Gónzalez-Hernández et al., 2020). Taking into account our previous results, we interrogated DEGs from our RNA-Seq data against recognized venous and coronary artery markers (Gónzalez-Hernández et al., 2020; Su et al., 2018). We found downregulation of *Apln* and overexpression of various venous markers, such as *Col1a1*, *Col3a1*, *Cpe*, *Dcn* and *Rcn3* (Fig. S6C). Expression of other canonical venous signatures, such as *Tek*, *Nrp2*, *Aplnr*, *Aqp1*, *Dab2*, *Ephb4*, *Emcn* and *Nr2f2*, did not change significantly (Table S2). To interrogate the distribution of venous ECs, immunofluorescence staining was performed at E18.5 on *Wt1KO^ΔEC^* hearts to visualize EMCN^+^ ECs, which displayed sub-epicardial localization in control E18.5 hearts. Quantitative analysis showed that the percentage of EMCN^+^ staining in the compact myocardium was increased in *Wt1KO^ΔEC^* mice (Fig. 6A,B).

Given that loss of *Wt1* impairs venous differentiation, it is to be expected that arterialization might also be affected. Changes in arterial markers were also modulated in the RNA-Seq data, as we observed a downregulation of *Rgs5*, *St8sia6*, *Nos2*, *Tox2*, *Epas1*, *Notch4* and *Jag2*, and an upregulation of *Hes1* and *Igfbp3* (Fig. S6C and Table S2). Other arterial genes such as *Slc45a4*, *Kcnj8*, *Gpc4*, *Gja4*, *Gja5*, *Cxcl12*, *Unc5b*, *Mecom*, *Nrp1*, *Sat1*, *Ptp4a3*, *Dll4*, *Hey1*, *Cxcr4*, *Chst1* and *Efnb2* did not show significant changes in expression (Table S2). To examine the distribution of mature arterial ECs, which display mid-myocardial localization in E18.5 control hearts, we performed immunofluorescence staining against the VMSC marker SMA and the arterial marker CX40. ECs surrounded by SMA^+^ VSMCs were scarce in the compact myocardium (Fig. 6C). Compared with control, CX40^+^CD31^+^ vessels in the *Wt1KO^ΔEC^* appeared as discontinuous patches of cells with much smaller lumens (Fig. 6D). Quantification of CD31^+^CX40^+^ vessels within the compact myocardium revealed a significant reduction in artery numbers in the *Wt1KO^ΔEC^* heart (Fig. 6E). Altogether, these findings demonstrate that deletion of *Wt1* in coronary ECs alters both the venous and arterial identity.

### Abnormal coronary artery development after late *Wt1* deletion in ECs

**Fig. 7.**
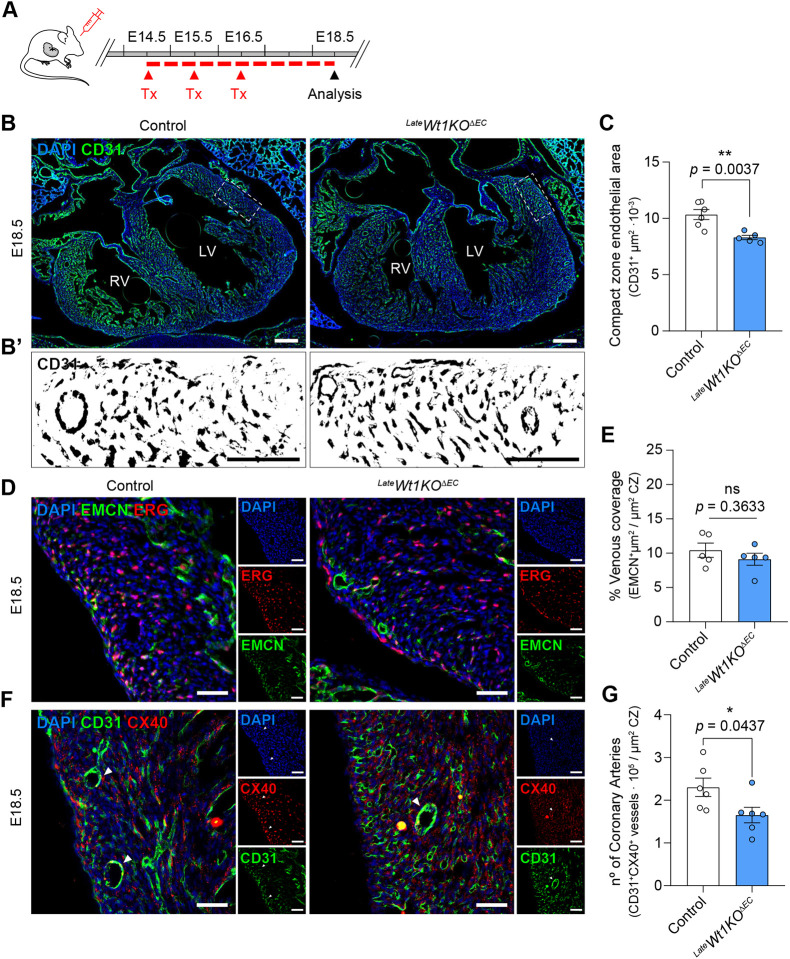
**Late *Wt1* deletion in coronary ECs affects coronary artery development.** (A) Schematic illustration showing the experimental protocol strategy to obtain and analyse a late *Wt1KO^ΔEC^* (*^Late^Wt1KO^ΔEC^)* mouse model. Pregnant mice were administered tamoxifen from E14.5 to E16.5 and embryos were analysed at E18.5. (B) Immunofluorescent staining for CD31 (green) and nuclear DAPI (blue), using heart sections from control and *^Late^Wt1KO^ΔEC^* at E18.5. (B′) Magnified images of CD31 staining from the areas outlined in B. (C) Quantitation of the CD31^+^ signal in the compact myocardium zone (CZ) reveals a reduction in the endothelial area of *^Late^Wt1KO^ΔEC^* hearts. Data are mean±s.e.m. *n*=5 or 6. *P<0.05 (unpaired *t*-test). (D) Immunostaining for endomucin (EMCN, green), ERG (red) and DAPI (blue) on E18.5 heart sections from control and *^Late^Wt1KO^EC^* mice. (E) Quantitation of EMCN^+^ signal in the CZ shows no alteration in venous coverage in *^Late^Wt1KO^EC^* hearts. Data are mean±s.e.m. *n*=5. ns: *P*≥0.05 (unpaired *t*-test). (F) Immunofluorescent staining of heart section from E18.5 hearts with antibodies against CD31 (green) and CX40 (red). (G) Quantitation of CX40^+^CD31^+^ vessels within the CZ reveals a reduction of arteries in the *^Late^Wt1KO^ΔEC^*. Data are mean±s.e.m. *n*=6. *P<0.05 (unpaired *t*-test). Scale bars: 250 µm in B; 100 µm in B′; 50 µm in D,F.

Given the requirement of WT1 during the initial phases of coronary plexus formation, we next analysed the effect of *Wt1* deletion during more mature phases, by administering tamoxifen from E14.5 to E16.5 (Fig. 7A and Fig. S8A). CD31 staining of this late *Wt1KO^ΔEC^* mouse model (*^Late^Wt1KO^ΔEC^*) at E18.5 demonstrated diminished coronary vascular density in the *^Late^Wt1KO^ΔEC^* compact myocardium zone of the heart (Fig. 7B,C). However, in contrast to the early model in which both venous and arterial specification were affected, ECM and CX40 staining in these *^Late^Wt1KO^ΔEC^* mice demonstrated that only the arterial differentiation was altered, supporting an additional role for WT1 in the regulation of coronary blood vessel development (Fig. 7D-G). On the other hand, quantification of compact and trabecular myocardium showed a decrease in the left ventricle compact myocardium zone in *^Late^Wt1KO^ΔEC^* hearts, and a small trabecular myocardium compensation in the same ventricle. However, these differences were not significant (Fig. S8B-D). Altogether, these data demonstrate that WT1 is a crucial gene for the embryonic development of coronary ECs and continues to play an important role beyond the early formation of the coronary plexus.

## DISCUSSION

The identification of new genes and pathways involved in physiological blood vessel formation is indispensable for the development of new therapies that promote angiogenesis after MI (Lupu et al., 2020). Here, using an inducible EC-specific *Wt1KO* mouse model, we demonstrate that WT1 in ECs is required for coronary angiogenesis and allows coronary vessels to support myocardial growth. Our study also provides strong evidence of WT1 as major regulator of the signature of coronary ECs, as the transcriptomic profile of coronary ECs from *Wt1KO^ΔEC^* mice revealed the dysregulation of crucial genes that are modulated over the course of coronary EC development (Gónzalez-Hernández et al., 2020).

Since the initial characterization of conventional *Wt1KO* mice, cardiovascular biologists have known that *Wt1* is a crucial gene for heart development (Moore et al., 1999). Nevertheless, most of the cardiovascular defects observed in *Wt1KO* mouse models with defects in heart development have mainly been attributed to WT1 functions in the epicardium and EPDCs (Guadix et al., 2011; Martinez-Estrada et al., 2010; Moore et al., 1999; Velecela et al., 2013, 2019; von Gise et al., 2011). Over the past two decades, several studies have demonstrated the expression of WT1 in coronary ECs during heart development and after MI (Duim et al., 2015; Wagner et al., 2002). In addition, recent studies have elegantly combined reporter mouse models with single-cell transcriptomic approaches, unequivocally demonstrating the endogenous expression of *Wt1* in coronary ECs during development and after MI (Forte et al., 2020). Despite the importance of these findings and their putative consequences for heart development and repair, little was known about the role of WT1 in coronary ECs. The analysis of the *Wt1KO^ΔEC^* mutant mice generated in this study reveals that conditional deletion of *Wt1* in ECs during the initial coronary angiogenic phase impairs the proliferation, migration and differentiation of immature coronary ECs and their subsequent remodeling into coronary arteries and veins. In *Wt1KO^ΔEC^* mice we observed defects in compact myocardium development that correlate with an expansion of the trabecular myocardium. The early lethality of the conventional *Wt1KO* mouse model has probably precluded the observation of this phenotype before (Kreidberg et al., 1993; Moore et al., 1999). As multiple mouse models with defective coronary vessel development display abnormal growth of the myocardium, we hypothesize that the myocardial defects observed in *Wt1KO^ΔEC^* mice might be due to defective signals from coronary ECs of the growing coronary plexus (D'Amato et al., 2016; Rhee et al., 2018; Travisano et al., 2019). In the more mature stages, restricted growth of the mutant myocardium may also be due to a combination of a reduction in angiocrine signals and the low diffusion capacity of oxygen and nutrients produced by a defective vasculature. Further studies are now needed to evaluate the frequency of *Wt1KO^ΔEC^* mice in the adult population.

Combining the transcriptomic data generated in this study with the transcriptomic profile of developing coronary ECs recently published by González-Hernández et al. has unequivocally demonstrated that WT1 expression exerts a major impact on the molecular signature of coronary ECs (Gónzalez-Hernández et al., 2020). During coronary blood vessel development, ECs derived from the SV lose their venous endothelial identity while gradually increasing the expression of arterial vessels (Su et al., 2018). In coronary ECs from *Wt1KO^ΔEC^* mice, we found a downregulation of several genes that are upregulated over the course of coronary blood vessel development, including arterial genes. We also observed the sustained expression of genes that should be downregulated over the course of coronary EC maturation. We reasoned that this gene signature demonstrates that mutant ECs remain in a more undifferentiated state and are unable to gradually increase the expression of genes from later developmental stages. In *Wt1KO^ΔEC^* mice, the expression of *Sox17*, *Dach1* or *N2rf2*, which are important players in coronary EC differentiation, is not affected, which suggests that WT1 directly controls key events of coronary blood vessel development independent of these factors (Chang et al., 2017; Gónzalez-Hernández et al., 2020; You et al., 2005). Among the many genes identified from the RNA-Seq analysis, *Foxo1*, *Meox2* and *Ets1* attracted our attention as they play an essential role in EC biology (Coppiello et al., 2015; Wilhelm et al., 2016; Wythe et al., 2013). It is therefore possible that some effects of WT1 on coronary ECs are dependent on their regulation, as some of these genes have been identified as WT1 targets (Motamedi et al., 2014). Although it would be very interesting to determine which of these genes are direct targets of WT1, the low number of ECs present in these embryonic hearts precludes ChIP sequencing analysis at these developmental stages.

In mouse, WT1 is expressed in ECs at different stages of coronary blood vessel development. In an attempt to determine whether WT1 expression is required for coronary EC development beyond the early phases of coronary plexus formation, we additionally generated a *^Late^Wt1KO^ΔEC^* mouse model. Interestingly, deletion of *Wt1* in ECs from E14.5 onwards, when the coronary plexus is already formed and pre-artery specification takes place, also led to a decrease in the coronary vascular density and defects in arterial differentiation. These findings demonstrate that WT1 is involved in several steps of coronary blood vessel development.

Here, we have shown for the first time that WT1 is required for physiological blood vessel formation in ECs and that its deletion during coronary plexus formation compromises myocardial development. The transcriptomic profile reported in this study constitutes an excellent resource for identifying new genes and pathways that are involved in the development of coronary ECs, as many of them are modulated during the course of this process. These findings have important implications for heart development and support further investigation of the role of WT1 in ECs in the revascularization of ischemic hearts.

## MATERIALS AND METHODS

### Animal models

The *Pdgfb^iCreERT2^, Wt1^LoxP^*, *R26^mTmG^* and UBC-Cre-ERT2 mice have been described previously (Claxton et al., 2008; Martinez-Estrada et al., 2010; Muzumdar et al., 2007; Ruzankina et al., 2007). In this study, we generated *Wt1^LoxP/+^*; *Pdgfb^iCreERT2/+^* and *Wt1^LoxP/LoxP^; Pdgfb^iCreERT2/+^* males, and *Wt1^LoxP/+^; R26^mTmG/mTmG^* and *Wt1^LoxP/LoxP^; R26^mTmG/mTmG^* females. Mating between *Wt1^LoxP/LoxP^; Pdgfb^iCreERT2/+^* males and *Wt1^LoxP/LoxP^* females allowed us to generate *Wt1^LoxP/LoxP^; Pdgfb^iCreERT2/+^* (*Wt1KO^ΔEC^*) and *Wt1^LoxP/LoxP^; Pdgfb^+/+^* (control) embryos. A second breeding strategy was followed by crossing *Wt1^LoxP/+^; Pdgfb^iCreERT2/+^* males with *Wt1^LoxP/+^; R26^mTmG/mTmG^* females to obtain *Wt1^LoxP/LoxP^; Pdgfb^iCreERT2^; R26^mTmG/+^* (*Wt1KO^ΔEC^*) and *Wt1^+/+^; Pdgfb^iCreERT2^; R26^mTmG/+^* (*Control^ΔEC^*) embryos from the same litter. Embryos were generated through timed matings, whereby females that had been mated overnight were checked for plugs early the following morning. The morning on which a plug was found was considered to be E0.5. Genotyping was carried out using DNA extracted from tail tip or ear biopsies. The primers used for genotyping are listed in Table S5. All animal experiments were carried out in accordance with the regulations of the Animal Experimentation Ethics Committee (CEEA) of the University of Barcelona (ID: PH3BYSQCC), thereby complying with current Spanish and European legislation.

### Tamoxifen dose

To induce Cre recombination, tamoxifen (Sigma-Aldrich, T5648) was dissolved in corn oil (Sigma-Aldrich, C8267) and administered at a dose of 75 mg tamoxifen/kg mouse weight. For pregnant mice, a stock of 20 mg tamoxifen/ml corn oil was administered through oral gavage (o.g.) by means of a stainless-steel feeding needle.

### Tissue processing and antibody staining

Mouse embryos obtained at the indicated stages were fixed with 4% paraformaldehyde (PFA) at 4°C for 2 h to overnight according to developmental stage. Depending on the immunostaining procedure, embryos were prepared for paraffin wax-embedded or frozen sections. Paraffin wax-embedded embryos were cut into 7 μm sections, deparaffinized in xylene and rehydrated with a serial ethanol gradient. For frozen sections, embryos were soaked in 15% and 30% sucrose sequentially, embedded in Tissue-Tek O.C.T. compound (Sakura) and frozen at −80°C. Embryos were then sectioned at 10 μm, mounted onto Superfrost Plus slides (Thermo Fisher Scientific) and stored at −80°C. For paraffin wax-embedded sections, an antigen retrieval procedure was carried out by boiling the samples in a pressure cooker for 15 min in citrate buffer (10 mM tri-sodium citrate 2-hydrate; pH 6.0); for cryosections, the antigen-retrieval step was carried out by boiling the samples at 60°C for 15 min in citrate buffer. The slides were then incubated in blocking serum [2% fetal bovine serum (FBS), 1% bovine serum albumin (BSA) in PBS with 0.1% Triton-X-100 (PBST)] for 2 h before being incubated overnight with the primary antibody at 4°C (see list of primary antibodies in Table S6). The slides were then washed with PBST, incubated at room temperature for 2 h with the appropriate secondary antibodies (listed in Table S7), stained with DAPI (Thermo Fisher Scientific, 62249) for 5 min for nuclear staining, and finally mounted with Fluoromount mounting medium (Sigma-Aldrich).

For immunofluorescent staining of whole-mount embryonic hearts, hearts were isolated in ice-cold PBS and immediately fixed in 4% PFA for 1 h at 4°C. Hearts were then permeabilized with PBST-0.5% for 1 h at room temperature, followed by incubation with blocking solution for at least 2 h at room temperature. Primary antibody incubation was performed for 48 h, at 4°C, with gentle rocking (see list of primary antibodies in Table S6). Hearts were then washed for 1 h with PBST-0.5%, four times at room temperature. Secondary antibody solution was incubated overnight, at 4°C, with gentle rocking (see Table S7). Four 1 h-washes with 0.5% PBST were performed at room temperature, and finally hearts were oriented (ventral/dorsal side facing down) in a glass-bottomed dish and immobilized with 1% agarose.

### BrdU injection and staining

To determine the number of proliferating cells, time-mated pregnant females were injected intraperitoneally with BrdU (Roche) solution (0.1 mg BrdU/kg mouse weight dose diluted in PBS) 2 h before they were euthanised. For complete DNA denaturation and exposure of the halogenated antigen, tissue sections were refixed with 4% PFA for 15 min, followed by a permeabilization step with 0.5% PBST. Sections were incubated with 2 N HCl solution for 60 min at 37°C, followed by incubation with 0.1 M boric acid (pH 8.5) for 30 min and PBS washes. Samples were incubated with blocking solution and the immunostaining procedure was followed, as previously described, using anti-BrdU (BD Biosciences, 3475-80; 1:100) and anti-BrdU (Abcam, ab6326; 1:100) primary antibodies (Table S6).

### Flow cytometry and cell sorting

To isolate and profile coronary ECs, heart ventricles were digested in 1 ml of digestion solution (1 mg/ml type 1 collagenase, Worthington, in HBSS Ca^2+^ Mg^2+^) for 20 min on a thermoblock with constant agitation (37°C, 1000 rpm). Collagenase activity was stopped by washing the cells in DMEM containing 5% of inactivated FBS. Finally, cells were pelleted by centrifugation and resuspended in HBSS Ca^2+^ Mg^2+^. FACS analysis was carried out using a FACS Aria III (BD Biosciences), and data were analysed using FACSDiva software (BD Biosciences). Cell gating of the corresponding transgenic fluorescent protein was carried out using samples negative for the fluorescent transgenic protein and isotype control antibodies.

### RNA isolation, cDNA synthesis and real-time qPCR

For RNA extraction from heart ventricles, the PureLink RNA Mini Kit (Invitrogen) was used according to the manufacturer's instructions. For RNA extraction from FACS-sorted cells, the RNeasy Micro Kit (Qiagen) was used according to the manufacturer's instructions. Purified RNA was used for reverse transcription and cDNA generation with SuperScript III Reverse Transcriptase (Thermo Fisher Scientific, 18080044).

TaqMan multiplex qRT-PCR was performed using FAM probes from the Universal ProbeLibrary (Roche Applied Science): 27 (for *Wt1 exon 1-2*) in combination with reference gene VIC probes and primers for β-actin. qRT-PCRs were performed using 96-well QuantStudio 3 with Thermo Fisher Connect analysis software.

### Low-input RNA-Seq

Samples were sequenced at CNAG-CRG (Barcelona, Spain). For RNA-Seq analysis, RNA from three biological replicates of FACS-isolated coronary ECs (∼8.000 cells) from *Control^ΔEC^* and *Wt1KO^ΔEC^* hearts were used to prepare low-input RNA sample sequencing libraries. RNA-Seq libraries were prepared by following the SMARTseq2 protocol with some modifications (Picelli et al., 2014). Briefly, RNA was quantified using the Qubit RNA HS Assay Kit (Thermo Fisher Scientific). Reverse transcription with the input material of 2 ng was performed using SuperScript II (Invitrogen) in the presence of oligo-dT30VN (1 µM; 5′-AAGCAGTGGTATCAACGCAGAGTACT30VN-3′), template-switching oligonucleotides (1 µM) and betaine (1 M). The cDNA was amplified using the KAPA Hifi Hotstart ReadyMix (Roche), 100 nM ISPCR primer (5′-AAGCAGTGGTATCAACGCAGAGT-3′) and 12 cycles of amplification. After purification with Agencourt Ampure XP beads (1:1 ratio; Beckmann Coulter), product size distribution and quantity were assessed on a Bioanalyser High Sensitvity DNA Kit (Agilent). The amplified cDNA (200 ng) was fragmented for 10 min at 55°C using Nextera XT (Illumina) and amplified for 12 cycles with indexed Nextera PCR primers. The library was purified twice with Agencourt Ampure XP beads (0.8:1 ratio) and quantified on a Bioanalyser using a High Sensitvity DNA Kit.

The libraries were sequenced on a NovaSeq 6000 (Illumina) in paired-end with a read length of 2×51bp according to the manufacturer's protocol for dual indexing. Image analysis, base calling and quality scoring of the run are processed using the manufacturer's software Real Time Analysis (RTA 3.4.4) and followed by generation of FASTQ sequence files.

### RNA-Seq data processing and analysis

Reads were mapped to the *Mus musculus* reference genome (GRCm39) using STAR/2.7.8a with ENCODE parameters. Gene quantification was performed with RSEM/1.3.0 using the gencode.vM27 version with default parameters. Differential expression analysis was carried out using the DESeq2 v1.18 R package with default parameters. Genes with Log2FC>|0.58| and *P*<0.05 were considered differentially expressed. Fold-change values between genotypes (*Wt1KO^ΔEC^* over *Control^ΔEC^*) are expressed in Log2FC (listed in Table S2). Gene Ontology (GO) enrichment analysis of these DEGs was performed using the ClueGO plug-in from Cytoscape software and the GO Biological Process database (version: EBI-UniProt-GOA-ACAP-ARAP_26.05.2022). GO Terms were obtained using a two tailed hypergeometric test corrected with a Bonferroni step down; GO Term Grouping *P*-values were also obtained using a two tailed hypergeometric test corrected with a Bonferroni step down using ClueGO kappa score (κ>0.4) to define term-term inter-relations and functional groups based on genes shared between terms, with the leading group term based on highest significance (listed in Table S3).

### Quantification of compact myocardium thickness

Comparable sections clearly displaying both atrioventricular valves were selected for the analysis. To delimit compact myocardium, immunostaining against MF20 (labelling all the myocardium) and EMCN (labelling the endocardium) was performed; the MF20^+^ EMCN^−^ region was considered the compact myocardium, whereas the MF20^+^ EMCN^+^ region was considered to be the trabecular myocardium. Composite pictures of whole-heart sections were obtained from partially overlapping images (Olympus BX61, 10× lens), using the ‘Grid/collection stitching’ function of ImageJ software. A minimum of six measures were taken at regular intervals and the average compact myocardial/trabecular thickness per ventricle was used to compare different genotypes.

### Quantification of immunofluorescence signal area and localization

High-resolution confocal images (Zeiss LSM 880, 40× lens, 2.048×2.048 pixels) with thin *z*-sectioning (0.5 µm) were taken. Evaluation of CD31^+^, ERG^+^, EMCN^+^ and CX40^+^ cell localization starting from the epicardium was performed using ImageJ software. DAPI counterstaining was used to delimit the epicardial layer, defined as the outer layer of nuclei. Each channel was adjusted for brightness and contrast, and filtered to obtain a mask. The staining profile was measured using the Plot profile function, and data values were adjusted to the maximum value section to obtain a staining percentage. For each embryo, a minimum of five image fields of the compact myocardium were quantified to obtain an average value.

### Quantification of endothelial polarity

Nuclear polarity was determined after immunostaining with the endothelial-specific nuclear protein marker ERG and counterstaining with DAPI to visualize nuclei. High-resolution confocal images (Zeiss LSM 880, 40× lens, 2.048×2.048 pixels) with thin *z*-sectioning (0.5 µm) were taken, and quantitation of nuclear dimensions of ERG^+^ cells was performed using ImageJ. To measure EC nuclei, scans of ERG and DAPI labelling were colocalized using the ‘Colocalization threshold’ function of ImageJ software. Subsequently, images were filtered to a threshold to obtain a binary image that was watershed, and images were analysed using the Analyse Particles function. Nuclear dimensions were evaluated via the Feret's Diameter function, and the nuclear length-to-width ratio was determined by dividing the Feret value by the minimum Feret for each cell. For each heart, at least four fields of view were assessed, and the average length-to-width ratio for each sample was used to compare different genotypes.

### Generation of tamoxifen-inducible *Wt1KO* endothelial cells

Lungs from P7 *Wt1^LoxP/LoxP^; UBC-CreERT2* mice were digested with type 1 collagenase (Worthington; 1 mg/ml) for 1 h at 37°C, with mild shaking every 2-5 min. The collagenase was inactivated by adding DMEM containing 10% of inactivated FBS. The cell suspensions were filtrated through a 100 µm cell strainer and pelleted by centrifugation (5 min, 1200 rpm). The dissociated cells were washed twice with PBS/BSA 0.5% followed by positive selection with anti-mouse VE-cadherin (Pharmingen, 555289) coated with magnetic beads for 30 min (Invitrogen, 11035). Purified cells were seeded on plates coated with gelatin plates (0.5%) in complete Endothelial Cell Growth Medium 2 (EGM-2; which is EBM-2 medium supplemented with EGM-2 SingleQuots). Confluent ECs were re-purified with anti-mouse VE-cadherin-coated magnetic beads. To induce *Wt1* deletion, 4-hydroxytamoxifen (4-OHT, 1 µM, Sigma-Aldrich, H7904) or vehicle (ethanol) was added to the culture medium. All the experiments were performed 72 h after the addition of 4-OHT or vehicle, and in ECs obtained immediately after the second round of purification.

### Transwell migration assay

EC migration was determined using a transwell migration assay. Briefly, control and *Wt1KO* ECs were cultured in a serum-free EBM-2 medium overnight before the assay. Trypsinized cells were seeded on gelatin-coated transwell membranes with 8 µm pores (Corning, 351152) at a density of 40,000 cells per well and allowed to migrate toward EGM-2 medium containing 2% FBS over a 16 h. After cell migration, solutions in the basal chamber were removed and basal membranes were washed twice with PBS. Next, 900 µl of 5 µM calcein solution (Invitrogen, C3100MP) dissolved in EBM-2 were added to the basal chamber and left to incubate at 37°C for 30 min. Calcein solution was washed and transwells were transferred to a new 24-well plate, maintaining the seeding solution in the bottom chamber until calcein fluorescence was read by measuring the 480/530nm emission on an Infinite 200 plate reader.

### BrdU proliferation assay

EC proliferation was quantified using a BrdU incorporated colorimetric cell proliferation ELISA assay (Roche Diagnostics). Briefly, 5000 control and *Wt1KO* cells were plated in gelatin-coated 96-well culture plates, with four replicas per condition. After 8 h of seeding, cells were synchronized in a serum-free EBM-2 medium. Cells were then incubated with BrdU labelling solution for another 24 h at 37°C in complete EBM-2 medium followed by fixation and incubation with anti-BrdU peroxidase conjugate for an additional 1.5 h at room temperature. Finally, after substrate reaction, colour intensity was measured with an Infinite 200 plate reader at 450 nm (reference wavelength: 690 nm).

### Statistical analysis

Data are presented as individual values and mean±s.e.m. Statistical significance between two groups was determined using an unpaired two-tailed Student's *t*-test. Statistical significance between cultured ECs was determined using a paired two-tailed Student's *t*-test. We applied non-parametric two-way ANOVA followed by Tukey's post-hoc test to evaluate differences among multiple groups of samples. For immunofluorescent signal localization data, significance was determined using a two-tailed Mann–Whitney test. GraphPad Prism 8 was used to generate graphs and for statistical analysis.

## Supplementary Material

10.1242/develop.201147_sup1 Supplementary information

## References

[DEV201147C1] Ariza, L., Rojas, A., Muñoz-Chápuli, R. and Carmona, R. (2019). The Wilms’ tumor suppressor gene regulates pancreas homeostasis and repair. *PLoS Genet.* 15, e1007971. 10.1371/journal.pgen.1007971 30763305 PMC6392337

[DEV201147C2] Bharathavikru, R., Dudnakova, T., Aitken, S., Slight, J., Artibani, M., Hohenstein, P., Tollervey, D. and Hastie, N. (2017). Transcription factor Wilms’ tumor 1 regulates developmental RNAs through 3′ UTR interaction. *Genes Dev.* 31, 347-352. 10.1101/gad.291500.116 28289143 PMC5358755

[DEV201147C3] Cano, E., Carmona, R., Ruiz-Villalba, A., Rojas, A., Chau, Y. Y., Wagner, K. D., Wagner, N., Hastie, N. D., Munoz-Chapuli, R. and Perez-Pomares, J. M. (2016). Extracardiac septum transversum/proepicardial endothelial cells pattern embryonic coronary arterio-venous connections. *Proc. Natl. Acad. Sci. U.S.A.* 113, 656-661. 10.1073/pnas.1509834113 26739565 PMC4725486

[DEV201147C4] Chang, A. H., Raftrey, B. C., D'amato, G., Surya, V. N., Poduri, A., Chen, H. I., Goldstone, A. B., Woo, J., Fuller, G. G., Dunn, A. R. et al. (2017). DACH1 stimulates shear stress-guided endothelial cell migration and coronary artery growth through the CXCL12-CXCR4 signaling axis. *Genes Dev.* 31, 1308-1324. 10.1101/gad.301549.117 28779009 PMC5580653

[DEV201147C5] Chau, Y. Y., Brownstein, D., Mjoseng, H., Lee, W. C., Buza-Vidas, N., Nerlov, C., Jacobsen, S. E., Perry, P., Berry, R., Thornburn, A. et al. (2011). Acute multiple organ failure in adult mice deleted for the developmental regulator Wt1. *PLoS Genet.* 7, e1002404. 10.1371/journal.pgen.1002404 22216009 PMC3245305

[DEV201147C6] Claxton, S., Kostourou, V., Jadeja, S., Chambon, P., Hodivala-Dilke, K. and Fruttiger, M. (2008). Efficient, inducible Cre-recombinase activation in vascular endothelium. *Genesis* 46, 74-80. 10.1002/dvg.20367 18257043

[DEV201147C7] Coppiello, G., Collantes, M., Sirerol-Piquer, M. S., Vandenwijngaert, S., Schoors, S., Swinnen, M., Vandersmissen, I., Herijgers, P., Topal, B., Van Loon, J. et al. (2015). Meox2/Tcf15 heterodimers program the heart capillary endothelium for cardiac fatty acid uptake. *Circulation* 131, 815-826. 10.1161/CIRCULATIONAHA.114.013721 25561514

[DEV201147C8] D'Amato, G., Luxan, G., del Monte-Nieto, G., Martínez-Poveda, B., Torroja, C., Walter, W., Bochter, M. S., Benedito, R., Cole, S., Martinez, F. et al. (2016). Sequential Notch activation regulates ventricular chamber development. *Nat. Cell Biol.* 18, 7-20. 10.1038/ncb3280 26641715 PMC4816493

[DEV201147C9] Dejana, E., Hirschi, K. K. and Simons, M. (2017). The molecular basis of endothelial cell plasticity. *Nat. Commun.* 8, 14361. 10.1038/ncomms14361 28181491 PMC5309780

[DEV201147C10] Dube, K. N., Thomas, T. M., Munshaw, S., Rohling, M., Riley, P. R. and Smart, N. (2017). Recapitulation of developmental mechanisms to revascularize the ischemic heart. *JCI insight* 2, e96800. 10.1172/jci.insight.96800 29202457 PMC5752387

[DEV201147C11] Duim, S. N., Kurakula, K., Goumans, M. J. and Kruithof, B. P. (2015). Cardiac endothelial cells express Wilms’ tumor-1: Wt1 expression in the developing, adult and infarcted heart. *J. Mol. Cell. Cardiol.* 81, 127-135. 10.1016/j.yjmcc.2015.02.007 25681586

[DEV201147C12] Forte, E., Skelly, D. A., Chen, M., Daigle, S., Morelli, K. A., Hon, O., Philip, V. M., Costa, M. W., Rosenthal, N. A. and Furtado, M. B. (2020). Dynamic interstitial cell response during myocardial infarction predicts resilience to rupture in genetically diverse mice. *Cell Reports* 30, 3149-3163.e3146. 10.1016/j.celrep.2020.02.008 32130914 PMC7059115

[DEV201147C13] Franco, C. A., Jones, M. L., Bernabeu, M. O., Vion, A. C., Barbacena, P., Fan, J., Mathivet, T., Fonseca, C. G., Ragab, A., Yamaguchi, T. P. et al. (2016). Non-canonical Wnt signalling modulates the endothelial shear stress flow sensor in vascular remodelling. *eLife* 5, e07727. 10.7554/eLife.07727 26845523 PMC4798962

[DEV201147C14] Gónzalez-Hernández, S., Gómez, M. J., Sanchez-Cabo, F., Méndez-Ferrer, S., Muñoz-Cánoves, P. and Isern, J. (2020). Sox17 controls emergence and remodeling of nestin-expressing coronary vessels. *Circ. Res.* 127, e252-e270. 10.1161/CIRCRESAHA.120.317121 32921258

[DEV201147C15] Guadix, J. A., Ruiz-Villalba, A., Lettice, L., Velecela, V., Muñoz-Chápuli, R., Hastie, N. D., Pérez-Pomares, J. M. and Martínez-Estrada, O. M. (2011). Wt1 controls retinoic acid signalling in embryonic epicardium through transcriptional activation of Raldh2. *Development* 138, 1093-1097. 10.1242/dev.044594 21343363 PMC3042868

[DEV201147C16] Hastie, N. D. (2017). Wilms’ tumour 1 (WT1) in development, homeostasis and disease. *Development* 144, 2862-2872. 10.1242/dev.153163 28811308

[DEV201147C17] He, L., Huang, X., Kanisicak, O., Li, Y., Wang, Y., Li, Y., Pu, W., Liu, Q., Zhang, H., Tian, X. et al. (2017). Preexisting endothelial cells mediate cardiac neovascularization after injury. *J. Clin. Invest.* 127, 2968-2981. 10.1172/JCI93868 28650345 PMC5531398

[DEV201147C18] Kreidberg, J. A., Sariola, H., Loring, J. M., Maeda, M., Pelletier, J., Housman, D. and Jaenisch, R. (1993). WT-1 is required for early kidney development. *Cell* 74, 679-691. 10.1016/0092-8674(93)90515-R 8395349

[DEV201147C19] Li, Z., Solomonidis, E. G., Meloni, M., Taylor, R. S., Duffin, R., Dobie, R., Magalhaes, M. S., Henderson, B. E. P., Louwe, P. A., D'amico, G. et al. (2019). Single-cell transcriptome analyses reveal novel targets modulating cardiac neovascularization by resident endothelial cells following myocardial infarction. *Eur. Heart J.* 40, 2507-2520. 10.1093/eurheartj/ehz305 31162546 PMC6685329

[DEV201147C20] Lu, P., Wang, Y., Liu, Y., Wang, Y., Wu, B., Zheng, D., Harvey, R. P. and Zhou, B. (2021). Perinatal angiogenesis from pre-existing coronary vessels via DLL4-NOTCH1 signalling. *Nat. Cell Biol.* 23, 967-977. 10.1038/s41556-021-00747-1 34497373

[DEV201147C21] Luo, W., Garcia-Gonzalez, I., Fernández-Chacón, M., Casquero-Garcia, V., Sanchez-Muñoz, M. S., Muhleder, S., Garcia-Ortega, L., Andrade, J., Potente, M. and Benedito, R. (2021). Arterialization requires the timely suppression of cell growth. *Nature* 589, 437-441. 10.1038/s41586-020-3018-x 33299176 PMC7116692

[DEV201147C22] Lupu, I. E., De Val, S. and Smart, N. (2020). Coronary vessel formation in development and disease: mechanisms and insights for therapy. *Nature Reviews. Cardiology* 17, 790-806. 10.1038/s41569-020-0400-1 32587347

[DEV201147C23] Martinez-Estrada, O. M., Lettice, L. A., Essafi, A., Guadix, J. A., Slight, J., Velecela, V., Hall, E., Reichmann, J., Devenney, P. S., Hohenstein, P. et al. (2010). Wt1 is required for cardiovascular progenitor cell formation through transcriptional control of Snail and E-cadherin. *Nat. Genet.* 42, 89-93. 10.1038/ng.494 20023660 PMC2799392

[DEV201147C24] Moore, A. W., Mcinnes, L., Kreidberg, J., Hastie, N. D. and Schedl, A. (1999). YAC complementation shows a requirement for Wt1 in the development of epicardium, adrenal gland and throughout nephrogenesis. *Development* 126, 1845-1857. 10.1242/dev.126.9.1845 10101119

[DEV201147C25] Motamedi, F. J., Badro, D. A., Clarkson, M., Rita Lecca, M., Bradford, S. T., Buske, F. A., Saar, K., Hubner, N., Brandli, A. W. and Schedl, A. (2014). WT1 controls antagonistic FGF and BMP-pSMAD pathways in early renal progenitors. *Nat. Commun.* 5, 4444. 10.1038/ncomms5444 25031030

[DEV201147C26] Muzumdar, M. D., Tasic, B., Miyamichi, K., Li, L. and Luo, L. (2007). A global double-fluorescent Cre reporter mouse. *Genesis* 45, 593-605. 10.1002/dvg.20335 17868096

[DEV201147C27] Picelli, S., Faridani, O. R., Björklund, Å. K., Winberg, G., Sagasser, S. and Sandberg, R. (2014). Full-length RNA-seq from single cells using Smart-seq2. *Nat. Protoc.* 9, 171-181. 10.1038/nprot.2014.006 24385147

[DEV201147C28] Quijada, P., Trembley, M. A., Misra, A., Myers, J. A., Baker, C. D., Perez-Hernandez, M., Myers, J. R., Dirkx, R. A., Jr, Cohen, E. D., Delmar, M. et al. (2021). Coordination of endothelial cell positioning and fate specification by the epicardium. *Nat. Commun.* 12, 4155. 10.1038/s41467-021-24414-z 34230480 PMC8260743

[DEV201147C29] Red-Horse, K., Ueno, H., Weissman, I. L. and Krasnow, M. A. (2010). Coronary arteries form by developmental reprogramming of venous cells. *Nature* 464, 549-553. 10.1038/nature08873 20336138 PMC2924433

[DEV201147C30] Rhee, S., Chung, J. I., King, D. A., D'amato, G., Paik, D. T., Duan, A., Chang, A., Nagelberg, D., Sharma, B., Jeong, Y. et al. (2018). Endothelial deletion of Ino80 disrupts coronary angiogenesis and causes congenital heart disease. *Nat. Commun.* 9, 368. 10.1038/s41467-017-02796-3 29371594 PMC5785521

[DEV201147C31] Ruzankina, Y., Pinzon-Guzman, C., Asare, A., Ong, T., Pontano, L., Cotsarelis, G., Zediak, V. P., Velez, M., Bhandoola, A. and Brown, E. J. (2007). Deletion of the developmentally essential gene ATR in adult mice leads to age-related phenotypes and stem cell loss. *Cell Stem Cell* 1, 113-126. 10.1016/j.stem.2007.03.002 18371340 PMC2920603

[DEV201147C32] Su, T., Stanley, G., Sinha, R., D'amato, G., Das, S., Rhee, S., Chang, A. H., Poduri, A., Raftrey, B., Dinh, T. T. et al. (2018). Single-cell analysis of early progenitor cells that build coronary arteries. *Nature* 559, 356-362. 10.1038/s41586-018-0288-7 29973725 PMC6053322

[DEV201147C33] Travisano, S. I., Oliveira, V. L., Prados, B., Grego-Bessa, J., Pineiro-Sabaris, R., Bou, V., Gomez, M. J., Sanchez-Cabo, F., Macgrogan, D. and De La Pompa, J. L. (2019). Coronary arterial development is regulated by a Dll4-Jag1-EphrinB2 signaling cascade. *eLife* 8, e49977. 10.7554/eLife.49977 31789590 PMC6917494

[DEV201147C34] Velecela, V., Lettice, L. A., Chau, Y. Y., Slight, J., Berry, R. L., Thornburn, A., Gunst, Q. D., Van Den Hoff, M., Reina, M., Martinez, F. O. et al. (2013). WT1 regulates the expression of inhibitory chemokines during heart development. *Hum. Mol. Genet.* 22, 5083-5095. 10.1093/hmg/ddt358 23900076

[DEV201147C35] Velecela, V., Torres-Cano, A., Garcia-Melero, A., Ramiro-Pareta, M., Muller-Sanchez, C., Segarra-Mondejar, M., Chau, Y. Y., Campos-Bonilla, B., Reina, M., Soriano, F. X. et al. (2019). Epicardial cell shape and maturation are regulated by Wt1 via transcriptional control of Bmp4. *Development* 146, dev178723. 10.1242/dev.178723 31624071

[DEV201147C36] Von Gise, A., Zhou, B., Honor, L. B., Ma, Q., Petryk, A. and Pu, W. T. (2011). WT1 regulates epicardial epithelial to mesenchymal transition through beta-catenin and retinoic acid signaling pathways. *Dev. Biol.* 356, 421-431. 10.1016/j.ydbio.2011.05.668 21663736 PMC3147112

[DEV201147C37] Wagner, K. D., Wagner, N., Bondke, A., Nafz, B., Flemming, B., Theres, H. and Scholz, H. (2002). The Wilms’ tumor suppressor Wt1 is expressed in the coronary vasculature after myocardial infarction. *FASEB J.* 16, 1117-1119. 10.1096/fj.01-0986fje 12039855

[DEV201147C38] Wilhelm, K., Happel, K., Eelen, G., Schoors, S., Oellerich, M. F., Lim, R., Zimmermann, B., Aspalter, I. M., Franco, C. A., Boettger, T. et al. (2016). FOXO1 couples metabolic activity and growth state in the vascular endothelium. *Nature* 529, 216-220. 10.1038/nature16498 26735015 PMC5380221

[DEV201147C39] Wu, B., Zhang, Z., Lui, W., Chen, X., Wang, Y., Chamberlain, A. A., Moreno-Rodriguez, R. A., Markwald, R. R., O'rourke, B. P., Sharp, D. J. et al. (2012). Endocardial cells form the coronary arteries by angiogenesis through myocardial-endocardial VEGF signaling. *Cell* 151, 1083-1096. 10.1016/j.cell.2012.10.023 23178125 PMC3508471

[DEV201147C40] Wythe, J. D., Dang, L. T., Devine, W. P., Boudreau, E., Artap, S. T., He, D., Schachterle, W., Stainier, D. Y., Oettgen, P., Black, B. L. et al. (2013). ETS factors regulate Vegf-dependent arterial specification. *Dev. Cell* 26, 45-58. 10.1016/j.devcel.2013.06.007 23830865 PMC3754838

[DEV201147C41] You, L. R., Lin, F. J., Lee, C. T., Demayo, F. J., Tsai, M. J. and Tsai, S. Y. (2005). Suppression of Notch signalling by the COUP-TFII transcription factor regulates vein identity. *Nature* 435, 98-104. 10.1038/nature03511 15875024

