# Supplementary material for: Endothelial deletion of Wt1 disrupts coronary angiogenesis and myocardium development
Source: Development. 2023 Mar 27;150(6):dev201147. doi: 10.1242/dev.201147 (PMC10112914; doi:10.1242/dev.201147)
Supplement: Supplementary information [file develop-150-201147-s1.pdf]

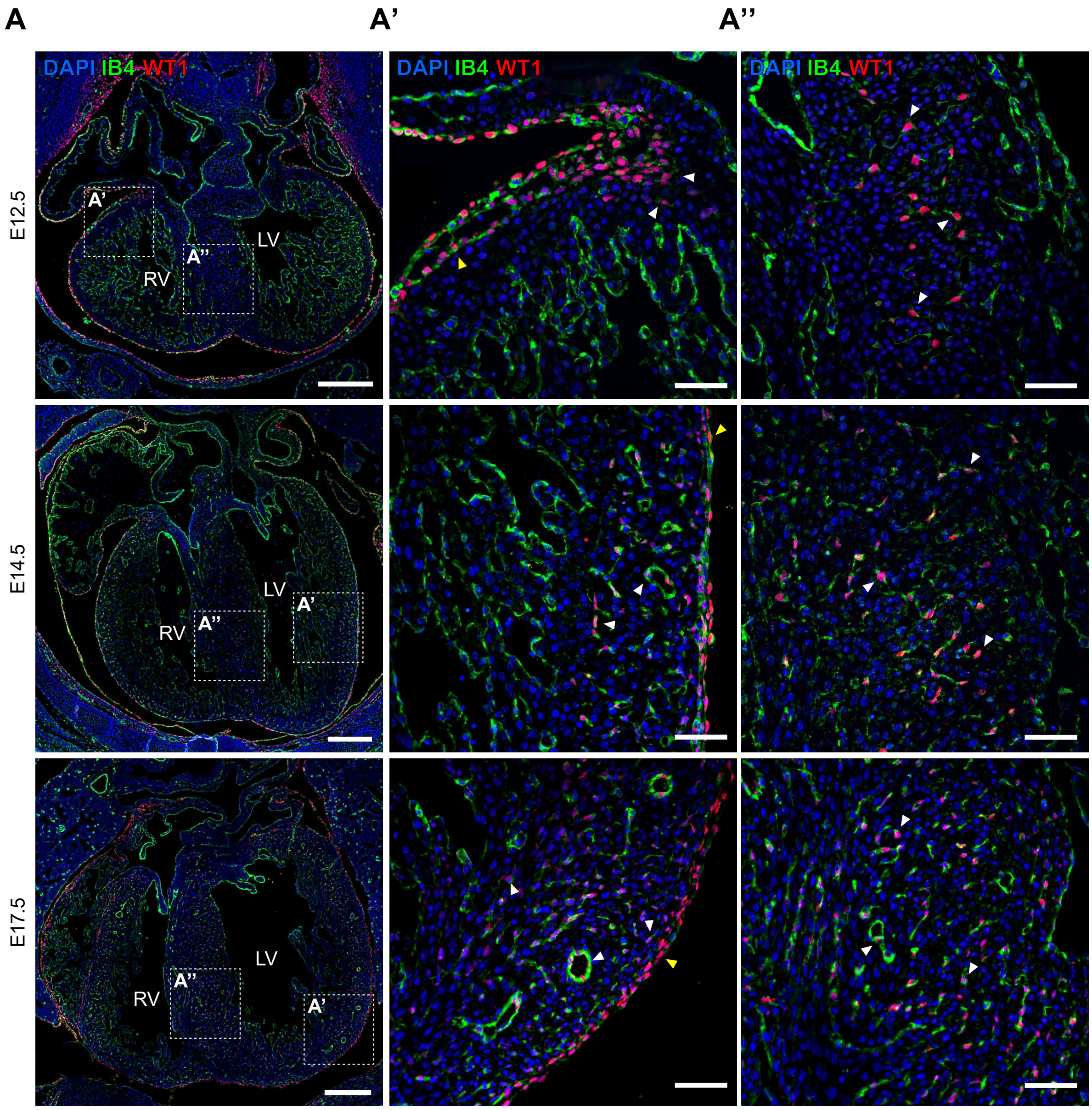

Fig. S1. WT1 expression in coronary ECs. (**A**) Immunostaining for WT1 (red) and staining of IB4 (green), and nuclear DAPI (blue) using heart sections at indicated developmental stages, and higher magnification images of the boxed areas (**A', A'')**. In addition to epicardium (yellow arrowheads) and EPDCs, WT1 expression is observed in ECs from the subepicardial plexus at E12.5 and in ECs from the interventricular septum (white arrowheads). By E14.5 and E17.5, WT1 expression can be observed in venous and arterial ECs from the coronary plexus, but not in the endocardium. Representative staining images of at least three embryos are shown. Scale bars: 250μm (A), 100μm (A', A'').

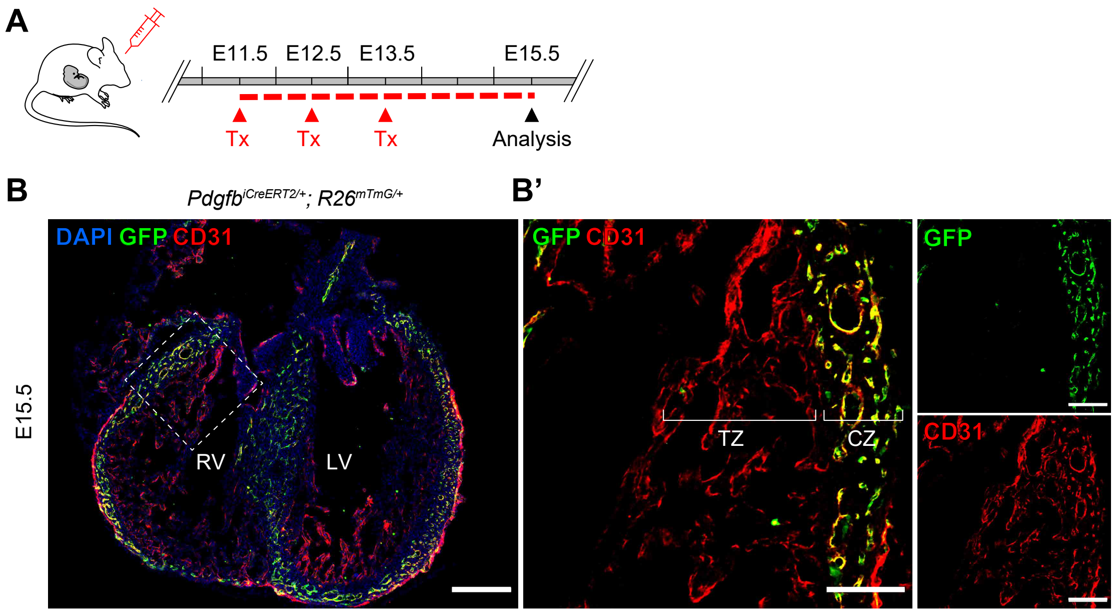

Fig. S2. *Pdgfb-iCreERT2* efficiently recombines in coronary ECs. (**A**) Schematic illustration showing the strategy to study the recombination of *Pdgfb-iCreERT2* line in coronary ECs. Pregnant mice carrying control and $Pdgfb^{iCreERT2/+}$; $R26^{mTmG/+}$ mice were administered tamoxifen during early stages of coronary formation onset (E11.5-E13.5) and embryos were analysed at E15.5. (**B, B'**) Immunostaining for CD31 (red), GFP (green), and nuclear DAPI staining (blue), using heart sections from $Pdgfb^{iCreERT2/+}$; $R26^{mTmG/+}$ E15.5 mice. A robust GFP expression is observed in coronary veins and arteries from the coronary plexus while no expression can be observed in the endocardium. Representative staining images of at least three embryos are shown. Scale bars: 250μm (B), 100μm (B').

Development • Supplementary information

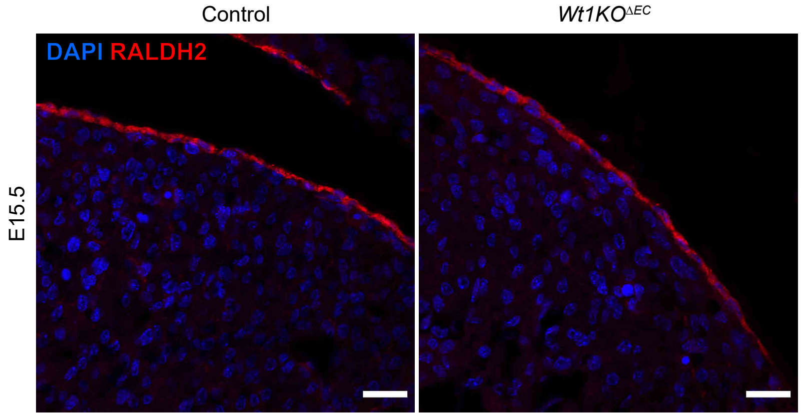

Fig. S3. Epicardium development is not affected in *Wt1KO*$^{\Delta EC}$ mice. Immunostaining for RALDH2 (red) and nuclear DAPI staining (blue), using heart sections from control and *Wt1KO*$^{\Delta EC}$ E15.5 mice. Representative staining images of at least three embryos are shown. Scale bar: 25μm.

Development • Supplementary information

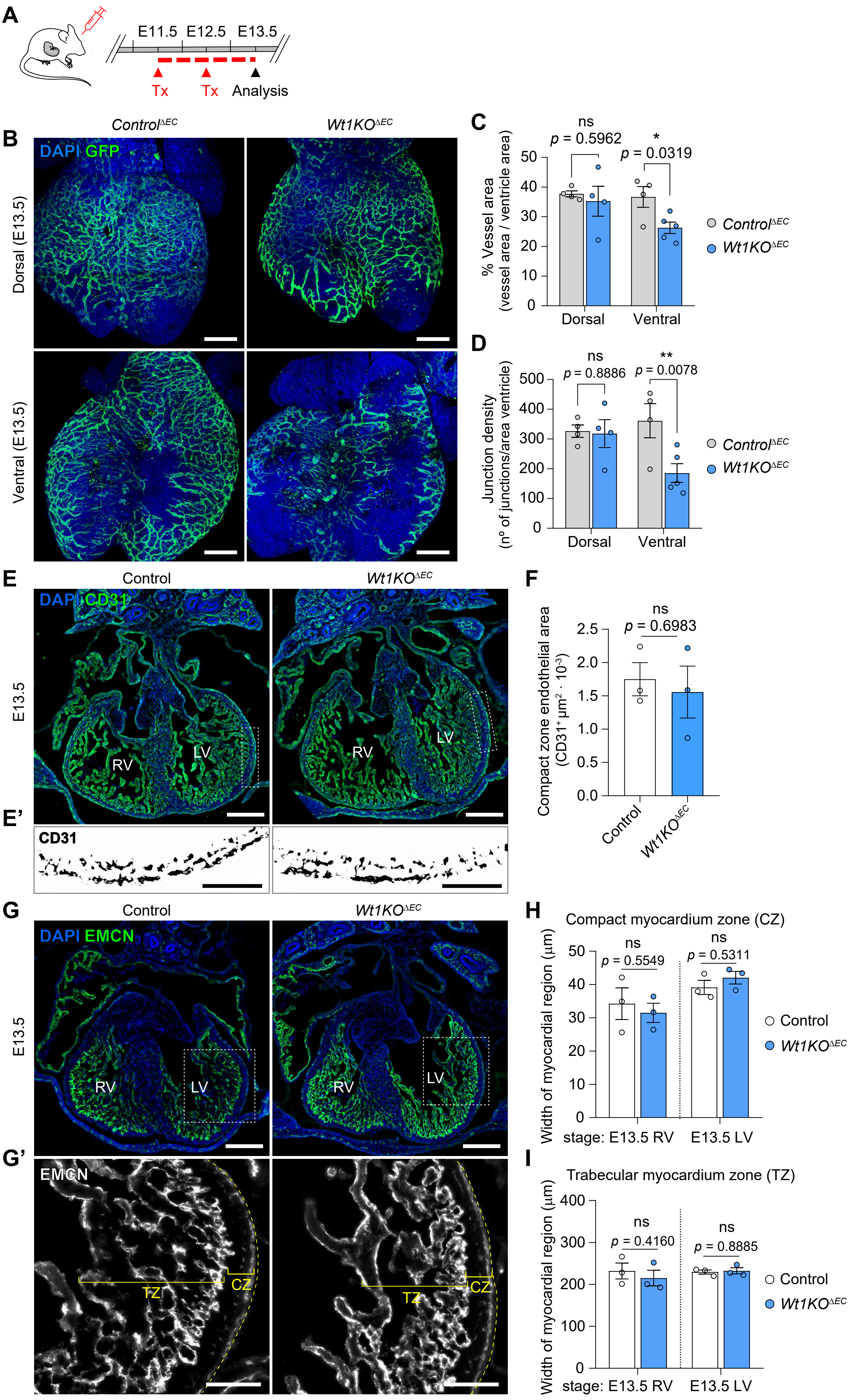

Development • Supplementary information

Fig. S4. Defects in coronary blood vessel development in *Wt1KO$^{\Delta EC}$* mice precede defects in myocardium development. (**A**) Schematic illustration showing the experimental protocol strategy to obtain and analyse *Wt1KO$^{\Delta EC}$* mice. Pregnant mice were administered tamoxifen from E11.5-E12.5 onward and embryos were analysed at E13.5. (**B**) Whole-mount immunofluorescence staining of GFP using hearts from *Control$^{\Delta EC}$* and *Wt1KO$^{\Delta EC}$* mice. (**C, D**) Quantification of vessel coverage and junctional density revealed a reduction in both parameters in the ventral side of *Wt1KO$^{\Delta EC}$* hearts. Values represent means ± s.e.m. (n=4-5). ns: $P \geq 0.05$, *$P<0.05$, **$P<0.005$, in a two-way ANOVA. (**E**) Immunostaining for CD31 (green) and nuclear DAPI staining (blue) using heart sections from Control and *Wt1KO$^{\Delta EC}$* mice at E13.5. (**E'**) Magnified images of CD31 staining in the compact myocardium zone from the areas boxed in E. (**F**) Quantitation of the CD31^{+} signal in the compact myocardium zone of control and *Wt1KO$^{\Delta EC}$* hearts. Values represent mean ± s.e.m. (n=3). ns: $P \geq 0.05$, unpaired t-test. (**G**) Immunostaining for Endomucin (EMCN, green) and nuclear DAPI staining (blue) on E13.5 heart sections from control and *Wt1KO$^{\Delta EC}$* mice. (**G'**) Boxed region in G, showing EMCN staining as an indicator of trabecular myocardium zone (TZ) vs. compact myocardium zone (CZ). (**H, I**) Quantification of CZ and TZ width in control and *Wt1KO$^{\Delta EC}$* mice. Values represent means ± s.e.m. (n=3). ns: $P \geq 0.05$, two-way ANOVA. Scale bars: 250µm (B, E, G) and 100µm (E' and G').

Development • Supplementary information

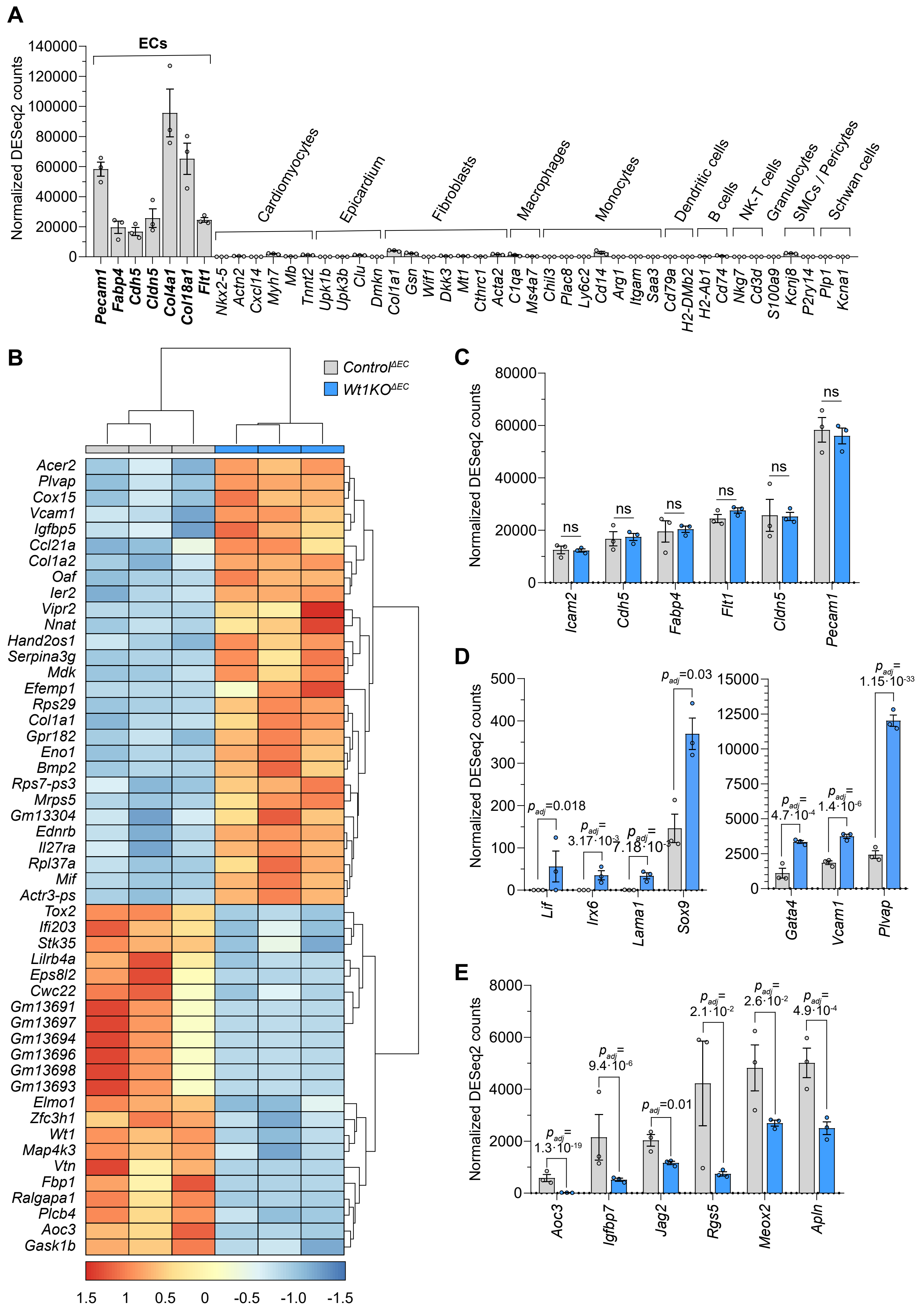
A
ECs
Normalized DESeq2 counts
140000
120000
100000
80000
60000
40000
20000
0
Cardiomyocytes
Epicardium
Fibroblasts
Macrophages
Monocytes
Dendritic cells
B cells
NK-T cells
Granulocytes
SMCs / Pericytes
Schwan cells
Pecam1
Fabp4
Cdh5
Cldn5
Col4a1
Col18a1
Flt1
Nkx2-5
Actn2
Cxcl14
Myh7
Mb
Tnnt2
Upk1b
Upk3b
Clu
Dmkn
Col1a1
Gsn
Wif1
Dkk3
Mt1
Cthrc1
Acta2
C1qa
Ms4a7
Chil3
Plac8
Ly6c2
Cd14
Arg1
Itgam
Saa3
Cd79a
H2-DMb2
H2-Ab1
Cd74
Nkg7
Cd3d
S100a9
Kcnj8
P2ry14
Plp1
Kcna1
B
ControlΔEC
Wt1KOΔEC
Acer2
Plvap
Cox15
Vcam1
Igfbp5
Ccl21a
Col1a2
Oaf
Ier2
Vipr2
Nnat
Hand2os1
Serpina3g
Mdk
Efemp1
Rps29
Col1a1
Gpr182
Eno1
Bmp2
Rps7-ps3
Mrps5
Gm13304
Ednrb
Il27ra
Rpl37a
Mif
Actr3-ps
Tox2
Ifi203
Stk35
Lilrb4a
Eps8l2
Cwc22
Gm13691
Gm13697
Gm13694
Gm13696
Gm13698
Gm13693
Elmo1
Zfc3h1
Wt1
Map4k3
Vtn
Fbp1
Ralgapa1
Plcb4
Aoc3
Gask1b
1.5
1
0.5
0
-0.5
-1.0
-1.5
C
Normalized DESeq2 counts
80000
60000
40000
20000
0
ns
Icam2
Cdh5
Fabp4
Flt1
Cldn5
Pecam1
D
Normalized DESeq2 counts
500
400
300
200
100
0
padj=0.018
padj= 3.17·10-3
padj= 7.18·10-5
padj=0.03
Lif
Irx6
Lama1
Sox9
15000
12500
10000
7500
5000
2500
0
padj= 4.7·10-4
padj= 1.4·10-6
padj= 1.15·10-33
Gata4
Vcam1
Plvap
E
Normalized DESeq2 counts
8000
6000
4000
2000
0
padj= 1.3·10-19
padj= 9.4·10-6
padj=0.01
padj= 2.1·10-2
padj= 2.6·10-2
padj= 4.9·10-4
Aoc3
Igfbp7
Jag2
Rgs5
Meox2
Apln

Fig. S5. Loss of *Wt1* in ECs modulates the gene signature of coronary ECs. (**A**) Normalized counts of gene expression of specific cellular signatures, showing over-representation of EC markers. (**B**) Heatmap of top 50 most significantly up- and downregulated genes in coronary ECs from *Control*$^{\Delta EC}$ vs. *Wt1KO*$^{\Delta EC}$ hearts. (**C**) Detailed comparison of expression levels for selected endothelial genes in *Control*$^{\Delta EC}$ and *Wt1KO*$^{\Delta EC}$ cells. (**D,E**) Detailed comparison of expression levels for selected DEGs that are also modulated over the course of coronary EC development in *Control*$^{\Delta EC}$ and *Wt1KO* $^{\Delta EC}$. Values represent mean ± s.e.m. from *Control*$^{\Delta EC}$ vs. *Wt1KO*$^{\Delta EC}$ hearts (n=3) normalized DESeq2 counts. Statistics correspond to the Log2FC p-value corrected with Benjamini-Hochberg procedure. P_{adj}<0.05.

Development • Supplementary information

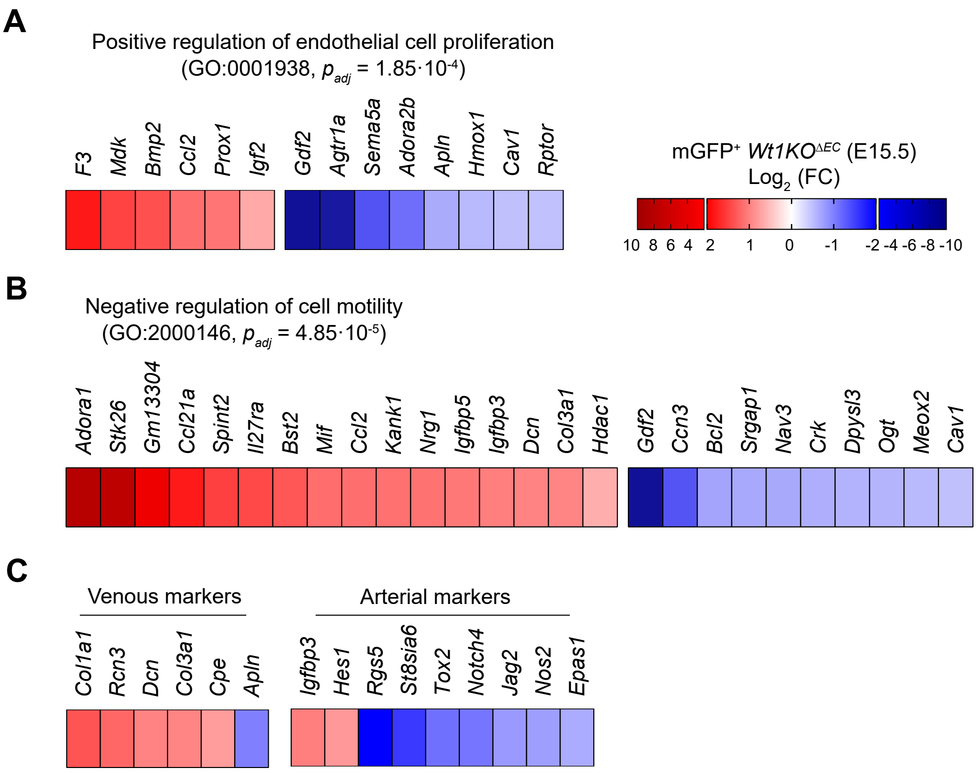

Fig. S6. Loss of *Wt1* in coronary ECs modulates the expression of genes involved in cell proliferation, motility and differentiation. Heatmaps constructed by selected genes belonging to the GO terms positive regulation of endothelial cell proliferation (**A**), negative regulation of cell motility (**B**) and by selected genes belonging to venous and arterial markers (**C**). GO Group p-values corrected with Bonferroni step down. $P_{adj}<0.05$.

Development • Supplementary information

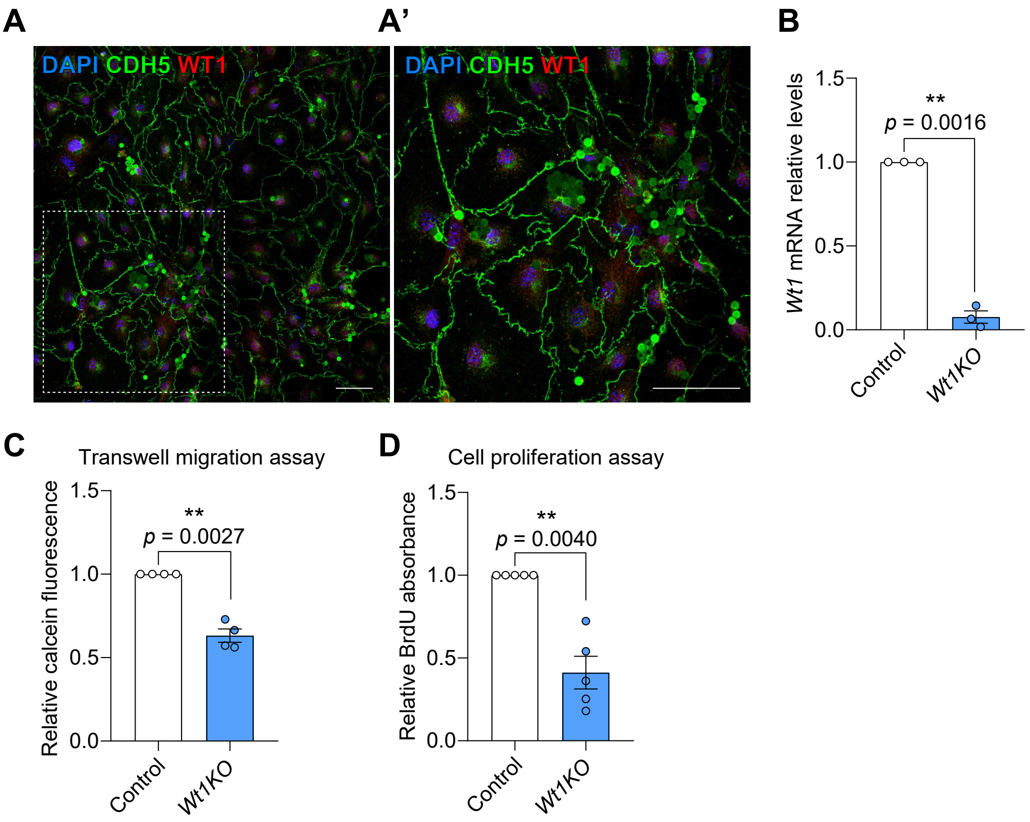

Fig. S7. In vitro deletion of *Wt1* impairs EC proliferation and migration. (**A**) Confocal immunofluorescence images of primary mouse ECs isolated from *$Wt1^{LoxP/LoxP}$; CreERT2* mice stained for CDH5 (green), WT1 (red) and nuclear DAPI staining (blue). (**A'**) Magnified images from the area boxed in A. (**B**) qRT-PCR analysis of *Wt1* expression after primary ECs being treated with vehicle control (Control) or 4-OHT (*Wt1KO*) for 72h. Values represent means ± s.e.m. (n=3). **P<0.005, paired two-tailed t-test. (**C**) Transwell migration assays of control and *Wt1KO* ECs, quantification of cell migration is presented as relative fluorescent units. **P<0.005, paired two-tailed t-test. (**D**) Proliferation assay using quantitative ELISA analysis of BrdU incorporation into control and *Wt1KO* ECs. Values represent means ± s.e.m. (n=5). **P<0.005, paired two-tailed t-test. Scale bars: 50µm.

Development • Supplementary information

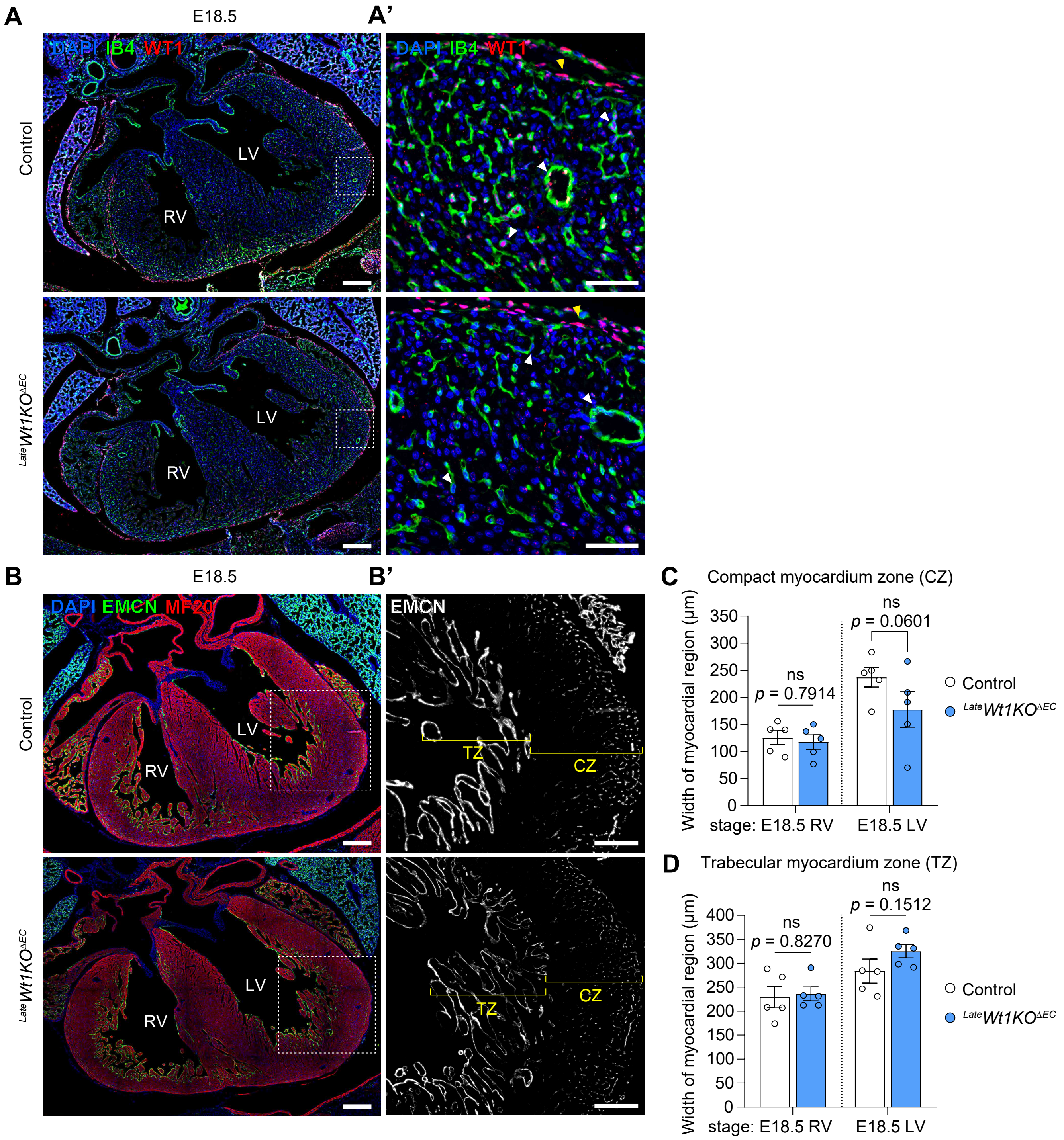

Fig. S8. Late deletion of *Wt1* in coronary ECs does not impair myocardium development. (**A**) Immunofluorescence staining for WT1 (red) and staining of IB4 (green) and nuclear DAPI (blue), using heart sections from control and $^{Late}Wt1KO^{\Delta EC}$ E18.5 mice. (**A'**) Magnification of boxed region in (A) showing specific downregulation of WT1 expression in coronary ECs (white arrowheads) while expression in the epicardium is not affected (yellow arrowheads). (**B**) Immunostaining for Endomucin (EMCN, green) and nuclear DAPI staining (blue) using heart sections from control and $^{Late}Wt1KO^{\Delta EC}$ mice at E18.5. (**B'**) Boxed region in B, showing EMCN staining as an indicator of trabecular myocardium zone (TZ) vs. compact myocardium zone (CZ). (**C, D**) Quantification of CZ and TZ width in control and $^{Late}Wt1KO^{\Delta EC}$ mice. Values represent means ± s.e.m. (n=5). ns: P≥0.05, two-way ANOVA. Scale bars: 250µm (A, B) and 50µm (A', B').

Development • Supplementary information

Table S1. *Wt1KO$^{\Delta EC}$* mice are not embryonic lethal. *Wt1KO$^{\Delta EC}$* ratios at different embryonic stages show a mendelian distribution of the genetic combinations resulting from the intercross of *Wt1$^{LoxP/LoxP}$; Pdgfb-iCreERT2* males with *Wt1$^{LoxP/ LoxP}$* females.

	**Control** (*Wt1$^{LoxP/LoxP}$*; *Pdgfb$^{+/+}$*)	***Wt1KO$^{\Delta EC}$*** (*Wt1$^{LoxP/LoxP}$*; *Pdgfb$^{iCreERT2/+}$*)
**E13.5**	15/26 (57.69%)	11/26 (42.31%)
**E15.5**	35/65 (53.85%)	30/65 (46.15%)
**E18.5**	8/16 (50.00%)	8/16 (50.00%)

Table S2. Differentially expressed genes in coronary ECs isolated from *Wt1KO$^{\Delta EC}$* vs *Control$^{\Delta EC}$* mice.

Click here to download Table S2

Table S3. List of GO Terms and Grouped GO terms based on the Gene Ontology Biological Process database.

Click here to download Table S3

Table S4. Differentially expressed genes in coronary ECs isolated from *Wt1KO$^{\Delta EC}$* vs *Control$^{\Delta EC}$* mice that are modulated over the course of coronary EC development.

Click here to download Table S4

Development • Supplementary information

Table S5. List of primers used for genotyping.

Gene	Primers (5'- … -3')	Program			PCR product
		Cycle	Temp	Time	
*Pdgfb^iCreERT2^*	**PDGFb_3UTR F:**<br>CCAGCCGCCGTCGCAACT<br>**PDGFb_3UTR R:**<br>GCCGCCGGGATCACTCTCG<br>**IL-2 F:**<br>CTAGGCCACAGAATTGAAAGATCT<br>**IL-2 R:**<br>GTAGGTGGAAATTCTAGCATCATC<br>C	1<br>34<br><br><br>1<br>1	94ºC<br>94ºC<br>57.5ºC<br>72ºC<br>72ºC<br>4ºC	3min<br>30s<br>45s<br>1min<br>1min<br>hold	Wild type:<br>300bp<br>Transgenic:<br>~430bp
*Wt1^LoxP^*	**Wt1-loxP F:**<br>TGGGTTCCAACCGTACCAAAGA	1<br>35	94ºC<br>94ºC	2min<br>15s	Wild type:<br>196bp
	**Wt1-loxP R:**<br>GGGCTTATCTCCTCCCATGT<br>**Wt1-loxP R2:**<br>GTACGCGCGAACACTGACTA	 <br>1<br>1	58ºC<br>72ºC<br>72ºC<br>4ºC	30s<br>1min<br>5min<br>hold	Transgenic:<br>230bp<br>Recombination band: ~500bp
*Rosa^mTmG^*	**Rosa10 F:**<br>CTCTGCTGCCTCCTGGCTTCT<br>**Rosa11_wt R:**<br>CGAGGCGGATCACAAGCAATA<br>**Rosa4_mTmG R:**<br>TCAATGGGCGGGGGTCGTT	1<br>35<br><br><br>1<br>1	94ºC<br>94ºC<br>58ºC<br>72ºC<br>72ºC<br>4ºC	2min<br>20s<br>25s<br>45s<br>5min<br>hold	Wild type:<br>330bp<br>Transgenic:<br>250bp

Development • Supplementary information

Table S6. List of primary antibodies used in this study.

Antibody/affinity molecule	Manufacturer	Cat. number	Species	Dilution	AR	BSA
Anti-ALDH1A2	Sigma-Aldrich	HPA010022	Rabbit	1:300	Yes	No
Anti-BrdU (B44)	BD	347580	Mouse	1:100	Yes	No
Anti-BrdU (BUI/75 (ICR)	Abcam	ab6326	Rat	1:100	Yes	No
Anti-CD31*	BD Pharmingen™	550274	Rat	1:100	Yes	No
Anti-CX40	Alpha Diagnostic International	CX40-A	Rabbit	1:100	Yes	Yes
Anti-Endomucin	Santa Cruz	sc-65495	Rat	1:100	Yes	No
Anti-ERG	Abcam	ab92513	Rabbit	1:300	Yes	No
Anti-GFP	Abcam	ab6673	Goat	1:300	Yes	No
Anti-MF20	DSHB	MF20-s	Mouse	1:50	No	No
Anti-αSMA	Cell Signalling	19245S	Rabbit	1:100	No	No
Anti-VE-cadherin	BD Pharmingen™	555289	Rat	1:100	No	No
Anti-WT1	Abcam	ab89901	Rabbit	1:100	Yes	Yes
Isolectin GS-IB4, AlexaFluor™ 488 conjugate	Invitrogen	I21411	-	1:50	No	No
Isolectin GS-IB4, biotinylated	Vector Laboratories	B-1205	-	1:50	No	Yes

AR: antigen retrieval, BSA: biotin signal amplification. *This antibody was used in frozen sections.

Development • Supplementary information

Table S7. List of secondary antibodies used in this study.

Antibody	Manufacturer	Cat. number	Species	Dilution
Anti-rat, AlexaFluor™ 488	Invitrogen	A11006	Goat	1:400
Anti-rat, AlexaFluor™ 546	Invitrogen	A11081	Goat	1:400
Anti-rat, AlexaFluor™ 633	Invitrogen	A21094	Goat	1:400
Anti-rabbit, AlexaFluor™ 488	Invitrogen	A21206	Donkey	1:400
Anti-rabbit, AlexaFluor™ 546	Invitrogen	A11035	Goat	1:400
Anti-rabbit, AlexaFluor™ 633	Invitrogen	A21070	Goat	1:400
Anti-goat, AlexaFluor™ 488	Invitrogen	A11055	Donkey	1:400
Anti-goat, AlexaFluor™ 633	Invitrogen	A21082	Donkey	1:400
Anti-mouse, AlexaFluor™ 488	Invitrogen	A21200	Chicken	1:400
Anti-mouse, AlexaFluor™ 546	Invitrogen	A11003	Goat	1:400
Biotin-SP-conjugated anti-Rabbit	Jackson	711-065-152	Donkey	1:400
Biotin-SP-conjugated anti-Goat	Jackson	715-065-147	Donkey	1:400
Biotin-SP-conjugated anti-Mouse	Jackson	715-065-150	Donkey	1:400
Streptavidin, AlexaFluor™ 488 conjugate	Invitrogen	S11223	-	1:400
Streptavidin, Cy™3 conjugate	Jackson	016-160-084	-	1:400
Streptavidin, AlexaFluor™ 647 conjugate	Invitrogen	S21374	-	1:400

Development • Supplementary information
