# Supplementary material for: Endothelial deletion of Wt1 disrupts coronary angiogenesis and myocardium development
Source: Development. 2023 Mar 27;150(6):dev201147. doi: 10.1242/dev.201147 (PMC10112914; doi:10.1242/dev.201147)
Supplement: Supplementary file 1 [file develop-150-201147_review_history.pdf]

Endothelial deletion of *Wt1* disrupts coronary angiogenesis and myocardium development

Marina Ramiro-Pareta, Claudia Müller-Sanchez, Rosa Portella-Fortuny, Carolina Soler-Botija, Alejo Torres-Cano, Anna Esteve-Codina, Antoni Bayés-Genis, Manuel Reina, Francesc X. Soriano, Eloi Montanez and Ofelia M. Martínez-Estrada
DOI: 10.1242/dev.201147

Editor: James Wells

Review timeline
Original submission: 22 July 2022
Editorial decision: 22 August 2022
First revision received: 31 January 2023
Accepted: 13 February 2023

Original submission

First decision letter

MS ID#: DEVELOP/2022/201147

MS TITLE: Endothelial deletion of Wt1 disrupts coronary angiogenesis and myocardium development

AUTHORS: Marina Ramiro-Pareta, Rosa Portella-Fortuny, Claudia Muller-Sanchez, Carolina Soler-Botija, Alejo Torres-Cano, Anna Esteve-Codina, Antoni Bayes-Genis, Manuel Reina, Francesc X. Soriano, Eloi Montanez, and Ofelia M Martinez-Estrada

I have now received all the referees' reports on the above manuscript, and have reached a decision. The referees' comments are appended below, or you can access them online: please go to BenchPress and click on the 'Manuscripts with Decisions' queue in the Author Area.

As you will see, the referees express considerable interest in your work, but have some significant criticisms and recommend a substantial revision of your manuscript before we can consider publication. If you are able to revise the manuscript along the lines suggested, which may involve further experiments, I will be happy receive a revised version of the manuscript. Your revised paper will be re-reviewed by one or more of the original referees, and acceptance of your manuscript will depend on your addressing satisfactorily the reviewers' major concerns. Please also note that Development will normally permit only one round of major revision. If it would be helpful, you are welcome to contact us to discuss your revision in greater detail. Please send us a point-by-point response indicating your plans for addressing the referee's comments, and we will look over this and provide further guidance.

Please attend to all of the reviewers' comments and ensure that you clearly highlight all changes made in the revised manuscript. Please avoid using 'Tracked changes' in Word files as these are lost in PDF conversion. I should be grateful if you would also provide a point-by-point response detailing how you have dealt with the points raised by the reviewers in the 'Response to Reviewers' box. If you do not agree with any of their criticisms or suggestions please explain clearly why this is so.

© 2023. Published by The Company of Biologists under the terms of the Creative Commons Attribution License (https://creativecommons.org/licenses/by/4.0/).

Reviewer 1

Advance summary and potential significance to field

In the manuscript by Ramiro-Pareta et al. the authors perform crosses to generate a line to examine the role of WT1 in endothelial cells in mice. Using an inducible Cre expressed in coronary ECs the authors provide experimental evidence of an uncharacterised functional role of WT1 in the coronary endothelium during embryonic cardiac development, as well in endothelial cells of the retina during post-natal stages. Although WT1 plays a critical role in the epicardium during heart development, this is the first report of a functional role for WT1 in coronary ECs.

In general, the authors are clear and concise and the conclusions are largely supported by the data, which is of a high quality. The previously unappreciated role for WT1 in endothelial development is novel and of interest for the field of cardiac development, as well as regeneration and vascular biology. While the findings are interesting, the link between WT1 and the coronary EC defect is missing a mechanistic explanation and the data provided on the role of WT1 in the retinal ECs lacks the care applied to the coronary EC model. Addressing the issues outlined in the comments below will help to strengthen the manuscript.

Comments for the author

Major issues:

1. Is the loss of WT1 in coronary endothelium lethal? This information is relatively simple to determine and should be reported in the manuscript.

2. In figure 3 the authors show a clear coronary EC phenotype at E15.5 after WT1 knock out at E11.5, however, in figure 4 the RNAseq at E15.5 revealed a large number of differentially expressed genes and it is impossible to distinguish between direct and indirect effects. Have earlier time points been examined to identify when the phenotype is first observed? In line with this, have the authors attempted a knock-out with constitutive endothelial/endocardial Cre lines (e.g. Tie2/Cdh5 and Nfatc1) to determine if any additional/earlier roles of WT1 in the coronary ECs/ endocardium exist?
From the RNAseq data, the authors mention that Foxo1, Meox2, and Ets1 “attracted our attention” (discussion, line 375), if there is evidence that these are direct targets of WT1 then ChIP qPCR on sorted ECs at the same timepoint when the defect is first observed would support such a hypothesis. Alternatively, ChIP-seq on sorted ECs would provide mechanistic insight that the current manuscript lacks.

3. Similar to the above point, in figure 2 the authors show a reduction in the compact layer myocardium at all timepoints examined, have earlier stages been examined to find when this reduction is first apparent? Presumably this follows some time after the EC defect first appears.

4. The GO term analysis in figure 4 lists muscle cell proliferation as one of the top hits, do the authors see a defect in CM proliferation or cell number that could explain the reduction in compact layer myocardium? The apparent decrease in EC number and decrease in compact layer myocardium suggests reduced proliferation/increased apoptosis occurs, yet this is at odds with the role of WT1 as a tumour suppressor, do the authors have an explanation for this?

5. Are the images in fig 5 and 6 taken from equivalent regions in control and mutant hearts? Please include images of the whole heart (either in the main figure or supplement) indicating this, as has been done in fig 2 and 3. Similarly, higher magnification images of the ERG positive nuclei would be useful to highlight the difference in nuclei shape observed in fig 5E.

6. In line 274 the authors state that “venous ECs displayed inappropriate accumulation near the epicardial surface” however the image shown appears to show an increase in endomucin staining throughout the compact layer, not just at the epicardial surface, as is also pointed out by the authors in line 276 and the quantification in fig 6B. It seems likely that this accumulation is caused by a general increase in endomucin staining throughout the compact layer and is not specific to the epicardial surface. As such I find the statement misleading, please consider rephrasing or removing it.

© 2023. Published by The Company of Biologists under the terms of the Creative Commons Attribution License (https://creativecommons.org/licenses/by/4.0/).

7. It does not seem that as much care was applied to the retina model as was afforded to the coronary EC model. Did the authors examine WT1 expression in the retina? If so, at what stages is WT1 expressed in the retina ECs? How widely expressed is the Pdgfb Cre line in retina ECs? How efficient is the knockdown of WT1 in these cells?
Further, while it is indeed important to report the EC phenotype observed in the retina during angiogenesis it is unclear how this data fits into the rest of the manuscript. Are the sprouting and angiogenesis defects observed in the retina also observed in the heart? Are the same genes that are differentially expressed in coronary ECs also altered in the retina ECs?
Additional experiments are required to bring this data to the same standard as the rest of the manuscript and highlight the similarities and differences between the role of WT1 in the two vascular beds.

Minor issues:
1. Line 63, "after the flow" should be written "after blood flow" or just "after flow"
2. Line 99, "as consequence" should be written "as a consequence"
3. Line 315, data mentioned refers to Fig. S7 not Fig. S6
4. Line 393, "in the heart development" should be written "in heart development"
5. Please consider reporting P-values in full in all figures to allow for more accurate interpretation of the results (see https://www.nature.com/articles/d41586-019-00857-9)

Reviewer 2

Advance summary and potential significance to field

Coronary endothelial cell proliferation and migration were deficient in Wt1 ablated hearts and the requirements for Wt1 in angiogenesis were confirmed in neonatal retinas.

Comments for the author

The manuscript by Ramiro-Pereta examines the requirements for Wt1 in coronary vessel development in mouse hearts. Conditional ablation of Wt1 using Pdgfb-iCre beginning at E11.5 leads to impaired cardiac vascular development and myocardial growth at E15.5. RNAseq shows alteration of endothelial gene expression and angiogenic processes. Coronary endothelial cell proliferation and migration were deficient in Wt1 ablated hearts and the requirements for Wt1 in angiogenesis were confirmed in neonatal retinas.

While the data demonstrating that Wt1 is required in coronary endothelial cells for normal vascular development are clear and well-presented, the specific functions of Wt1 in terms of downstream target genes, intersecting signaling pathways, and regulation of specific cellular processes in coronary endothelial cells were not examined in depth or demonstrated from the data presented.

Comments
1. The conditional ablation of Wt1 in coronary endothelial cells leads to severe abnormalities in the heart muscle and vasculature when examined at E15.5. Overall development of the heart seems to be delayed which may account for some of the differential expression of endothelial maturation genes and defective myocardial development. From the data presented, it is not clear what the direct and indirect effects of loss of Wt1 are on the developing heart.

2. There may be some clues to specific processes regulated by Wt1 in the RNAseq data. It is curious that several endothelial marker genes (Supp Fig 4C) were not differentially expressed in the knockout embryos while fibroblast marker Sox9 was increased. In addition some genes related to specific signaling pathways were affected. More in depth analysis is needed to sort out the direct and indirect target genes and determine what the specific functions of Wt1 are in coronary endothelial cells.

3. Much is known about various signaling pathways and gene hierarchies in angiogenesis. The analysis in the current study is superficial and does not determine the specific timing or developmental processes that require Wt1 in coronary vessel development.

© 2023. Published by The Company of Biologists under the terms of the Creative Commons Attribution License (https://creativecommons.org/licenses/by/4.0/).

Reviewer 3

Advance summary and potential significance to field

In this study Ramiro-Pareta et al. employ a conditional knockout mouse model to elucidate the role of the transcription factor Wt1 in cardiac endothelial cells.
Mid-gestational deletion of Wt1 in cardiac endothelium resulted in impaired development of the coronary blood vessels and a thinning of the myocardial compact zone. Transcriptional profiling of knockout cells revealed considerable alterations in gene expression, which were associated with phenotypes including reduced endothelial proliferation, reduced migration, and stunted differentiation. Examination of Wt1 function in retinal endothelial cells revealed similar results.

Wt1 is commonly regarded as an epicardial specific cardiac transcription factor.
This manuscript overturns that assumption by convincingly showing defects in development of the coronary arteries and myocardium following endothelial ablation. The paper is well-designed, highly focused, and well-written. The data appear to be of high quality, with proper effort given to making quantitative comparisons with application of appropriate statistical tests. Overall, this is a very nice manuscript; however, a couple issues should be addressed:

Comments for the author

Comments:
1. While the embryonic defects in coronary and myocardial development resulting from Wt1 endothelial KO are clear, as currently presented their significance is not. Do Wt1KOEC mice die? If so, when? If not, do the phenotypes go away? Do the mice have reduced cardiac function?
2. On line 251 the text says there's an inappropriate accumulation of ECs near the epicardial surface in Wt1KOEC hearts at E18.5, while Figure 5D seems to show that the inappropriate accumulation is at E15.5 and has normalized by E18.5. Perhaps the panels are switched?

Minor Comments:
1. The analysis of genes regulated by Wt1 would be greatly strengthen by having Wt1 ChIP-seq data for cross comparison with the transcriptional profiling. However, this reviewer understands the technical challenges of endothelial specific ChIP in embryonic hearts.
2. Using CM in figures to label the compact myocardium is a little confusing since CM means cardiomyocyte in most cardiac papers. I would suggest using CZ for compact zone.

First revision

Author response to reviewers' comments

Reviewer 1

Advance Summary and Potential Significance to Field:

In the manuscript by Ramiro-Pareta et al. the authors perform crosses to generate a line to examine the role of WT1 in endothelial cells in mice. Using an inducible Cre expressed in coronary ECs the authors provide experimental evidence of an uncharacterised functional role of WT1 in the coronary endothelium during embryonic cardiac development, as well in endothelial cells of the retina during post-natal stages. Although WT1 plays a critical role in the epicardium during heart development, this is the first report of a functional role for WT1 in coronary ECs.

In general, the authors are clear and concise and the conclusions are largely supported by the data, which is of a high quality. The previously unappreciated role for WT1 in endothelial development is novel and of interest for the field of cardiac development, as well as regeneration and vascular biology. While the findings are interesting, the link between WT1 and the coronary EC defect is missing a mechanistic explanation and the data provided on the role of WT1 in the retinal ECs lacks the care applied to the coronary EC model. Addressing the issues outlined in the comments below will help to strengthen the manuscript.

© 2023. Published by The Company of Biologists under the terms of the Creative Commons Attribution License (https://creativecommons.org/licenses/by/4.0/).

R: We sincerely appreciate the reviewer's critical reading of the manuscript and their constructive comments, which have helped us to introduce important improvements.

Reviewer 1 Comments for the Author:

Major issues:

1. Is the loss of WT1 in coronary endothelium lethal? This information is relatively simple to determine and should be reported in the manuscript.

R: The loss of WT1 in coronary ECs during coronary plexus formation is not embryonic lethal. This information is now included in Table S1.

2. In figure 3 the authors show a clear coronary EC phenotype at E15.5 after WT1 knock out at E11.5, however, in figure 4 the RNAseq at E15.5 revealed a large number of differentially expressed genes and it is impossible to distinguish between direct and indirect effects. Have earlier time points been examined to identify when the phenotype is first observed? In line with this, have the authors attempted a knock-out with constitutive endothelial/endocardial Cre lines (e.g. Tie2/Cdh5 and Nfatc1) to determine if any additional/earlier roles of WT1 in the coronary ECs/ endocardium exist?

R: Following the reviewer's suggestion, we examined coronary EC development in *Wt1KO*$^{\Delta EC}$ mice at E13.5. Whole-mount staining of GFP on hearts from Control$^{\Delta EC}$ and *Wt1KO*$^{\Delta EC}$ embryos demonstrated that the ventral coverage of the coronary plexus is affected at E13.5 in mutant embryos. These data are now included in Fig. S4 of the revised version of the manuscript.

Regarding the second point, we have not attempted to generate constitutive endothelial *Wt1KO* mouse models. We opted for a tamoxifen-inducible Cre driver and administered tamoxifen to delete the expression of *Wt1* from the earliest time point that we observed its expression in coronary ECs (Fig. S1 and Fig. 1).

From the RNAseq data, the authors mention that Foxo1, Meox2, and Ets1 "attracted our attention" (discussion, line 375), if there is evidence that these are direct targets of WT1 then ChIP qPCR on sorted ECs at the same timepoint when the defect is first observed would support such a hypothesis. Alternatively, ChIP-seq on sorted ECs would provide mechanistic insight that the current manuscript lacks.

R: Unfortunately, at present this type of experiment is very difficult to perform and technically challenging. Although ChIP and ChIPseq have been performed in cell lines and organs where WT1 is expressed, ChIP of WT1 on a low number of sorted cells is technically challenging. Isolating ECs at E13.5 yields around 2000 cells per embryonic heart and ChIP of a transcription factor usually uses around a million cells per replica (Kidder et al., 2011).

3. Similar to the above point, in figure 2 the authors show a reduction in the compact layer myocardium at all timepoints examined, have earlier stages been examined to find when this reduction is first apparent? Presumably this follows some time after the EC defect first appears.

R: We thank reviewer 1 for this suggestion. We have performed the experiment proposed and found that the myocardium development was not affected at E13.5. This information is now included in Fig. S4G-I.

4. The GO term analysis in figure 4 lists muscle cell proliferation as one of the top hits, do the authors see a defect in CM proliferation or cell number that could explain the reduction in compact layer myocardium? The apparent decrease in EC number and decrease in compact layer myocardium suggests reduced proliferation/increased apoptosis occurs, yet this is at odds with the role of WT1 as a tumour suppressor, do the authors have an explanation for this?

R: Following this reviewer's suggestion we assessed cardiomyocyte proliferation in control and *Wt1KO*$^{\Delta EC}$ embryos. Immunostaining for BrdU together with myosin heavy chain (MF20) revealed a

© 2023. Published by The Company of Biologists under the terms of the Creative Commons Attribution License (https://creativecommons.org/licenses/by/4.0/).

significant decrease in cardiomyocyte proliferation of E15.5 *Wt1KO*$^{\Delta EC}$ hearts. This information is included in Fig. 2E, F.

Regarding the second point, we are not sure that we have understood the question. Although *WT1* was identified as a gene mutated in a subset of Wilms' tumors, and classified as a tumor suppressor, in recent decades its natural role in the formation and homeostasis of several tissues has been demonstrated, meaning that it does not only act as a tumor suppressor (Hastie, 2017).

5. Are the images in fig 5 and 6 taken from equivalent regions in control and mutant hearts? Please include images of the whole heart (either in the main figure or supplement) indicating this, as has been done in fig 2 and 3. Similarly, higher magnification images of the ERG positive nuclei would be useful to highlight the difference in nuclei shape observed in fig 5E.

R: All the immunostaining data included in figures 5 and 6 were quantified and are represented in Fig. 5B, D, E, and Fig. 6 B, E. High-resolution confocal images were taken from equivalent regions in control and mutant hearts. For each embryo, a minimum of five images of the compact myocardium zone were quantified to obtain an average value; a minimum of three embryos were analysed. Providing lower magnification of the images included in Figs 5 and 6 is not possible, as these pictures were not taken when we performed the experiments. Higher magnification of the ERG positive nuclei is now included in Fig. 5C.

6. In line 274 the authors state that "venous ECs displayed inappropriate accumulation near the epicardial surface" however the image shown appears to show an increase in endomucin staining throughout the compact layer, not just at the epicardial surface, as is also pointed out by the authors in line 276 and the quantification in fig 6B. It seems likely that this accumulation is caused by a general increase in endomucin staining throughout the compact layer and is not specific to the epicardial surface. As such I find the statement misleading, please consider rephrasing or removing it.

R: We thank reviewer 1 for pointing this out. We have removed the sentence (line 302).

7. It does not seem that as much care was applied to the retina model as was afforded to the coronary EC model. Did the authors examine WT1 expression in the retina? If so, at what stages is WT1 expressed in the retina ECs? How widely expressed is the Pdgfb Cre line in retina ECs? How efficient is the knockdown of WT1 in these cells?

Further, while it is indeed important to report the EC phenotype observed in the retina during angiogenesis it is unclear how this data fits into the rest of the manuscript. Are the sprouting and angiogenesis defects observed in the retina also observed in the heart? Are the same genes that are differentially expressed in coronary ECs also altered in the retina ECs?

Additional experiments are required to bring this data to the same standard as the rest of the manuscript and highlight the similarities and differences between the role of WT1 in the two vascular beds.

R. In response to the reviewer's comments, we have removed the retina data from this revised version. We have focused our efforts on strengthening the rest of the data by generating 26 new panels related to the role of WT1 in coronary ECs.

Minor issues:

1. Line 63, "after the flow" should be written "after blood flow" or just "after flow"

R: We have corrected the sentence (line 63).

2. Line 99, "as consequence" should be written "as a consequence"

R: Corrected (line 99).

3. Line 315, data mentioned refers to Fig. S7 not Fig. S6

© 2023. Published by The Company of Biologists under the terms of the Creative Commons Attribution License (https://creativecommons.org/licenses/by/4.0/).

R: These data have been removed from the current version of the article.

4. Line 393, “in the heart development” should be written “in heart development”

R: Corrected (line 407).

5. Please consider reporting P-values in full in all figures to allow for more accurate interpretation of the results (see https://www.nature.com/articles/d41586-019-00857-9)

R: We thank the reviewer for this suggestion. We have modified the figures, and the revised version of the article includes P-values.

Reviewer 2

Advance Summary and Potential Significance to Field:

Coronary endothelial cell proliferation and migration were deficient in Wt1 ablated hearts and the requirements for Wt1 in angiogenesis were confirmed in neonatal retinas.

Reviewer 2 Comments for the Author:

The manuscript by Ramiro-Pereta examines the requirements for Wt1 in coronary vessel development in mouse hearts. Conditional ablation of Wt1 using Pdgfb-iCre beginning at E11.5 leads to impaired cardiac vascular development and myocardial growth at E15.5. RNAseq shows alteration of endothelial gene expression and angiogenic processes. Coronary endothelial cell proliferation and migration were deficient in Wt1 ablated hearts and the requirements for Wt1 in angiogenesis were confirmed in neonatal retinas. While the data demonstrating that Wt1 is required in coronary endothelial cells for normal vascular development are clear and well-presented, the specific functions of Wt1 in terms of downstream target genes, intersecting signaling pathways, and regulation of specific cellular processes in coronary endothelial cells were not examined in depth or demonstrated from the data presented.

R: We sincerely appreciate the reviewer’s critical reading of the manuscript and their comments.

Comments

1. The conditional ablation of Wt1 in coronary endothelial cells leads to severe abnormalities in the heart muscle and vasculature when examined at E15.5. Overall development of the heart seems to be delayed ,which may account for some of the differential expression of endothelial maturation genes and defective myocardial development. From the data presented, it is not clear what the direct and indirect effects of loss of Wt1 are on the developing heart.

R: In the initial version of the manuscript, in Figure 1C we showed a robust and specific deletion of *Wt1* in coronary ECs of *Wt1KO*$^{\Delta EC}$ mice. In this revised version, we have provided new data demonstrating that coronary blood vessel defects are present at E13.5 when the myocardium development is still not affected (Fig. S4). Thus, the myocardium defects observed in *Wt1KO*$^{\Delta EC}$ mice are indirect effects of WT1 loss in coronary ECs.

2. There may be some clues to specific processes regulated by Wt1 in the RNAseq data. It is curious that several endothelial marker genes (Supp Fig 4C) were not differentially expressed in the knockout embryos while fibroblast marker Sox9 was increased. In addition some genes related to specific signaling pathways were affected. More in depth analysis is needed to sort out the direct and indirect target genes and determine what the specific functions of Wt1 are in coronary endothelial cells.

R: During the revision process, we have generated new data that demonstrate that *in vitro* deletion of *Wt1* in endothelial cells impairs migration and proliferation in a cell- autonomous manner. This new information is included in Fig. S7 of the revised version.

© 2023. Published by The Company of Biologists under the terms of the Creative Commons Attribution License (https://creativecommons.org/licenses/by/4.0/).

3. Much is known about various signaling pathways and gene hierarchies in angiogenesis. The analysis in the current study is superficial and does not determine the specific timing or developmental processes that require Wt1 in coronary vessel development.

R: In comparison with other fields of vascular biology, the understanding of the signaling pathways and gene hierarchies involved in different aspects of coronary ECs is still limited (Trimm and Red-Horse, 2022). We believe our study represents an important step forward, in so far as it is the first to identify the requirement of WT1 functions in coronary ECs.

We specifically administered tamoxifen from E11.5 onward because, until this study, the abnormal cardiac vascularization and thinning of the ventricular myocardium observed in conventional *Wt1KO* mice have mainly been attributed to defects arising from the epicardium and EPDCs. It was not our intention to determine the specific time at which WT1 is necessary for correct coronary development. However, following this criticism, we extended the analysis and generated a late *Wt1KO*$^{\Delta EC}$ mouse model (^{Late}*Wt1KO*$^{\Delta EC}$). In contrast to the model in which *Wt1* was deleted during the coronary plexus formation and both venous and arterial identity were affected, in this ^{Late}*Wt1KO*$^{\Delta EC}$ just the arterial differentiation was altered, suggesting an additional role for WT1 in the regulation of coronary blood vessel development (Fig. 7).

Reviewer 3

Advance Summary and Potential Significance to Field:

In this study Ramiro-Pareta et al. employ a conditional knockout mouse model to elucidate the role of the transcription factor Wt1 in cardiac endothelial cells. Mid- gestational deletion of Wt1 in cardiac endothelium resulted in impaired development of the coronary blood vessels and a thinning of the myocardial compact zone. Transcriptional profiling of knockout cells revealed considerable alterations in gene expression, which were associated with phenotypes including reduced endothelial proliferation, reduced migration, and stunted differentiation. Examination of Wt1 function in retinal endothelial cells revealed similar results.
Wt1 is commonly regarded as an epicardial specific cardiac transcription factor. This manuscript overturns that assumption by convincingly showing defects in development of the coronary arteries and myocardium following endothelial ablation. The paper is well- designed, highly focused, and well-written. The data appear to be of high quality, with proper effort given to making quantitative comparisons with application of appropriate statistical tests. Overall, this is a very nice manuscript; however, a couple issues should be addressed:

R: We were pleased to read reviewer 3's very positive evaluation.

Reviewer 3 Comments for the Author:

Comments:

1. While the embryonic defects in coronary and myocardial development resulting from Wt1 endothelial KO are clear, as currently presented their significance is not. Do Wt1KOEC mice die? If so, when? If not, do the phenotypes go away? Do the mice have reduced cardiac function?

R: The *Wt1KO*$^{\Delta EC}$ model is not embryonic lethal, as we observed a normal mendelian ratio in all the embryonic stages analysed (Table S1), and the few mutant pups that we were able to analyse were alive (data not shown). The *Wt1KO*$^{\Delta EC}$ model is tamoxifen- inducible, and so we are not able to easily evaluate postnatal viability beyond P0, owing to the tamoxifen-associated effect in pregnant mice delivery.

If not, do the phenotypes go away? Do the mice have reduced cardiac function?

R: In this study, we analysed the effect of *Wt1* deletion on embryonic heart development. Although we are very interested in these questions raised by reviewer 3, this study would take some time to complete, given the effect of tamoxifen on pregnant mice delivery. It is very likely that the embryonic heart defects observed in *Wt1KO*$^{\Delta EC}$ compromise the heart functions of mutant mice,

© 2023. Published by The Company of Biologists under the terms of the Creative Commons Attribution License (https://creativecommons.org/licenses/by/4.0/).

because mouse models with thin myocardium phenotypes that survive usually display altered cardiac functions.

2. On line 251 the text says there's an inappropriate accumulation of ECs near the epicardial surface in Wt1KOEC hearts at E18.5, while Figure 5D seems to show that the inappropriate accumulation is at E15.5 and has normalized by E18.5. Perhaps the panels are switched?

R: We thank reviewer 3 for pointing this out. As the reviewer says, there is an inappropriate accumulation of ECs in $Wt1KO^{EC}$ hearts at E15.5. The sentence has been modified (line 271).

Minor Comments:

1. The analysis of genes regulated by Wt1 would be greatly strengthen by having Wt1 ChIP-seq data for cross comparison with the transcriptional profiling. However, this reviewer understands the technical challenges of endothelial specific ChIP in embryonic hearts.

R: We thank the reviewer for this observation, and we agree with the statement. ChIP of WT1 on a low number of sorted cells is technically challenging. Isolating ECs during embryonic stages yields around 2000-3000 cells per embryonic heart and ChIP of a transcription factor usually uses a million cells per replica (Kidder et al., 2011).

2. Using CM in figures to label the compact myocardium is a little confusing since CM means cardiomyocyte in most cardiac papers. I would suggest using CZ for compact zone.

R: We thank the reviewer for pointing this out. We have modified the figures.

Hastie, N. D. (2017). Wilms' tumour 1 (WT1) in development, homeostasis and disease. *Development* **144**, 2862-2872.
Kidder, B. L., Hu, G. and Zhao, K. (2011). ChIP-Seq: technical considerations for obtaining high- quality data. *Nature immunology* **12**, 918-922.
Trimm, E. and Red-Horse, K. (2022). Vascular endothelial cell development and diversity. *Nature reviews. Cardiology*.

Second decision letter

MS ID#: DEVELOP/2022/201147

MS TITLE: Endothelial deletion of Wt1 disrupts coronary angiogenesis and myocardium development

AUTHORS: Marina Ramiro-Pareta, Claudia Muller-Sanchez, Rosa Portella-Fortuny, Carolina Soler-Botija, Alejo Torres-Cano, Anna Esteve-Codina, Antoni Bayes-Genis, Manuel Reina, Francesc X. Soriano, Eloi Montanez, and Ofelia M Martinez-Estrada
ARTICLE TYPE: Research Article

I am happy to tell you that your manuscript has been accepted for publication in Development, pending our standard ethics checks.

Reviewer 1

Advance summary and potential significance to field

In the manuscript by Ramiro-Pareta et al. the authors perform crosses to generate a line to examine the role of WT1 in endothelial cells in mice. Using an inducible Cre expressed in coronary ECs the authors provide experimental evidence of an uncharacterised functional role of WT1 in the coronary endothelium during embryonic cardiac development. Although WT1 plays a critical role in

© 2023. Published by The Company of Biologists under the terms of the Creative Commons Attribution License (https://creativecommons.org/licenses/by/4.0/).

the epicardium during heart development, this is the first report of a functional role for WT1 in coronary ECs.

In general, the authors are clear and concise and the conclusions are supported by the data, which is of a high quality. The previously unappreciated role for WT1 in endothelial development is novel and of interest for the field of cardiac development, as well as regeneration and vascular biology.

Comments for the author

The authors have addressed most of my issues and I agree with the removal of the data from the retina model for the purposes of this manuscript. The only issue that has not been directly addressed is the lack of ChIP-seq to identify the direct targets of WT1 in coronary ECs. However, the authors point out there are technical challenges to performing such an experiment. This is an understandable limitation but I hope the authors consider performing this experiment in the future, perhaps low input ChIP-seq methods (e.g. PMID: 25607992) can help with this.
The revised manuscript is strengthened as a result of the changes that have been made, and is suitable for publication in Development.

Reviewer 2

Advance summary and potential significance to field

The manuscript by Ramiro-Pereta examines the requirements for Wt1 in coronary vessel development in mouse hearts. Conditional ablation of Wt1 using Pdgfb-iCre beginning at E11.5 leads to impaired cardiac vascular development and myocardial growth at E15.5. RNAseq shows alteration of endothelial gene expression and angiogenic processes. Coronary endothelial cell proliferation and migration were deficient in Wt1 ablated hearts.

Comments for the author

The revised manuscript now has more evidence for Wt1 requirements in endothelial maturation and migration.
The inclusion of earlier E13.5 data supports a primary effect in endocardial cells since the muscle is not affected at this time. Additional new data show that WT1 deficient endothelial cells have defective migration in cell culture.

Concerns regarding lack of information on direct interactions of WT1 with known endocardial signaling pathways or downstream target gene expression were not addressed. There do seem to be some interesting gene alterations in the RNAseq data shown in supplemental Figure 3, but these were not validated or examined in any depth in vivo. This information could be of use to researchers going forward.

Reviewer 3

Advance summary and potential significance to field

This revision further strengthens the previous conclusion that WT1 plays an important role in coronary angiogenesis by regulating genes involved in cell proliferation, migration, and differentiation.

Comments for the author

My previous comments have been adequately addressed.

© 2023. Published by The Company of Biologists under the terms of the Creative Commons Attribution License (https://creativecommons.org/licenses/by/4.0/).
